# Research on the Performance Prediction of Fly Ash Slurry by Neural Network Based on Information Bottleneck Theory and Attention Mechanism

**DOI:** 10.3390/ma19183929

**Published:** 2026-09-16

**Authors:** Botuan Deng, Zifan Li, Yujun Lai, Yixiang Feng, Bin Sun, Ruilin Zhang, Yunwei Bai

**Affiliations:** 1 School of Architecture and Civil Engineering, Xi’an University of Science and Technology, Xi’an 710054, China; 2 China State Construction Sixth Bureau Transportation Construction Co., Ltd., Hangzhou 311300, China

**Keywords:** fly ash slurry, information bottleneck theory, attention mechanism, slurry performance prediction, convolutional neural networks

## Abstract

**Highlights:**

- An IB-AM-CNN model predicts six fly ash slurry properties from three mix proportion variables.
- Dual optimization cuts mean error from 18.38% to 10.26%, a 44% improvement over baseline CNN.
- A model-derived recommended mix (1.25:1 w/b ratio, 20% fly ash, 10% water glass) was evaluated through field loading tests.

**Abstract:**

In grouting projects, the relationship between slurry properties and mix proportions is multivariate and nonlinear. Researchers have widely employed mathematical and computational methods to investigate this relationship, and the use of machine learning to predict the properties of fly ash slurries has gradually become a hot topic. To this end, this paper uses a convolutional neural network (CNN) to establish predictive models relating grout mix proportions to the properties of the grout and the resulting stone bodies. It investigates the effects of the water-to-binder ratio, fly ash dosage, and water glass volumetric dosage on the slurry density, viscosity, initial setting time, and final setting time of fly ash grout, as well as on the compressive strength and stone rate of the corresponding stone bodies. The CNN is optimized using the information bottleneck theory and attention mechanisms, and finally, based on nine sets of self-designed experiments, compares four neural network models for predicting fly ash performance. The results show that the information bottleneck–attention dual-optimized convolutional neural network had an average prediction error of 10.26%, which represents a 44% improvement over the average prediction error of 18.38% for a single convolutional neural network. It also achieved multi-objective performance prediction for fly ash slurry. Finally, the reliability of this prediction model was verified through a combination of laboratory and field tests.

## 1. Introduction

China’s comprehensive utilization of fly ash has reached 663 million tonnes in 2022. As a solid emission from thermal power plants, fly ash reuse can alleviate the pressure of overproduction in terms of the environment [1]; in recent years, fly ash has become a common admixture in concrete and grouting projects. Indeed, in China, the demand for fly ash in the construction industry accounts for 56 per cent of total fly ash production [2]. Fly ash is employed globally for engineering applications. The construction of the Hungry Horse Dam was made possible by using fly ash [3]. The use of fly-ash–rice-husk-ash blended concrete has been demonstrated to reduce CO_2_ greenhouse gas emissions [4]. El Mendili, Y [5] combines fly ash with French gravel and other waste materials to create a novel building material. The utilization of fly ash in numerous construction projects has been demonstrated to be a practical approach, with the incorporation of fly ash in engineering construction offering a means of conserving resources and safeguarding the environment. The resource-based utilization of fly ash aligns with global emissions reduction strategies and the requirements for green, low-carbon development, and serves as an important means of promoting the reduction, recycling, and efficient utilization of bulk industrial solid waste.

In recent years, scholars have conducted extensive research on the proportion of fly ash slurry, investigating the impact of slurry composition on performance. This research employs machine learning techniques, complementing traditional mathematical analysis methods. Previous studies have shown that fly ash can improve the performance of engineering materials—such as backfill, road subbases, concrete, and soil—when blended with various cementitious materials, solid waste, and modified materials; at the same time, machine learning methods can be used to establish predictive relationships between material mix proportions and engineering performance. He Xiang [6] employed a large-scale three-dimensional crushed gangue grouting filling test device approach to investigate the efficacy of fly ash slurries in grouting and filling operations. Huo, W [7] incorporated fly ash and ordinary Portland cement into a mixture of reclaimed lime-and-fly-ash crushed stone powder, and applied this mixture to road subbases or subgrades. Ashfaq, M. H. [8] added available kaolin to soil-polymer concrete using fly ash as a binder to improve its compressive strength and splitting strength. Hwang, S [9] studied the optimization of the proportions of seawater-mixed pervious concrete with substandard fly ash for compressive strength and permeability. Wasil and Zabielska-Adamska [10] added fly ash to bentonite to improve the plastic properties of the soil. Li, W et al. [11] used SHAP-based explainable machine learning to derive explicit prediction formulas from a black-box model of concrete carbonation, balancing accuracy and generalizability. Zhang, Z et al. [12] employed CTGAN data augmentation and BO-XGBoost to address the challenge of predicting UHPC performance with limited data, and used SHAP to reveal the mechanisms underlying the effects of mineral admixtures. Liu X et al. [13] systematically reviewed the progress and challenges in applying machine learning and deep learning algorithms to the prediction of the performance, hydration, and carbon emissions of low-carbon cementitious materials. Yeh et al. [14] addressed the subject of machine learning in predicting concrete strength for the first time. They employed an extensive database of artificial neural networks with seven input variables for predicting high-performance concrete strength. Wang X.Y. [15,16] used a genetic algorithm to identify the optimal cost-effective composition for slag fly ash concrete, with specific focus on the desired strength and service life. Some researchers [17,18,19] have employed artificial neural networks to forecast parameters such as the strength of concrete and to establish a predictive relationship between proportioning and concrete properties. Several researchers [20,21,22,23,24,25,26] have undertaken a comparative analysis of the Random Forest Algorithm (RF), Vector Machines (SVM) and artificial neural networks (ANN) to predict the relationship between concrete proportioning and performance. The results of this analysis have demonstrated that each of the algorithms above has a distinct effect on predicting different performances. Deng [27] trained a convolutional neural network using 74 datasets to establish a prediction model for concrete compressive strength. The results demonstrate that the convolutional neural network prediction model exhibits high accuracy and strong generalization ability. Other scholars [28,29] have conducted a comparative analysis of the prediction accuracy of compressive strength using a range of algorithms. The findings indicate that convolutional neural networks exhibit superior prediction performance compared to models such as gated recurrent units (GRUs) and long short-term memory (LSTM) networks.

Overall, existing research has gradually evolved from traditional empirical mix designs and mathematical analysis to the use of machine learning for predicting material properties and optimizing mix designs; however, the focus has largely been on concrete, and the predicted metrics have primarily centered on single properties such as compressive strength.

Based on the studies cited above, the effects of fly ash incorporation on the mix proportions and properties of grout have consistently attracted considerable research attention. Over the past two decades, many researchers have employed computer-aided analysis and machine learning techniques to optimize the mix designs of various types of concrete. However, research on grout for backfill applications has primarily focused on the compressive strength of the backfill material. In practical grouting projects, however, parameters such as grout flowability, setting behavior, the strength of the solidified mass, and the solidification rate must simultaneously satisfy engineering requirements. Therefore, intelligent machine learning methods are needed to predict and optimize multiple performance objectives for grout. This paper uses a convolutional neural network to predict the properties of fly ash slurry and innovatively employs the information bottleneck theory and attention mechanisms to improve the convolutional neural network model, thereby enhancing prediction accuracy.

## 2. Materials and Methods

In the grouting project, the performance of slurry directly affects the effect of grouting, and the impact of slurry on the project is generally used to evaluate the bearing capacity, precipitation, controllability and rheology. The compressive strength, as the primary evaluation parameter of bearing capacity and the most fundamental parameter of the grouting project, is investigated to ensure that the strength of the stone body is sufficient to meet the needs of the project. As the primary evaluation parameter of bleeding, the stone rate helps ensure that the strength of the stone body and the reinforcement effect meet the requirements; viscosity, as the primary evaluation parameter of rheology, is also significant, as it ensures that the slurry is not excessively lost or unable to flow. The setting time of the slurry directly determines the controllability of the grouting project; the controllable initial setting time makes the slurry not condense prematurely to block the equipment or flow channel in the process of grouting equipment and injection into the target area, and the controllable final setting time prevents the slurry from being lost excessively in the target area. At the same time, the premature final setting time may result in poor engagement of the slurry in the soil body, leading to slurry loss. At the same time, too early a final setting time may cause the slurry in the soil body engagement to not be good, resulting in the stone body and soil body not being coordinated with force deformation, resulting in a poor grouting effect. Therefore, the slurry density, viscosity, initial setting time and final setting time of the slurry, the compressive strength of the formed stone body and the stone rate are taken as the prediction objects to ensure the maximum closeness between the model and the project.

Fly ash slurry comprises water, cement, fly ash and water glass. This study employs the water-to-binder ratio of fly ash, the mass admixture of fly ash and the volumetric water glass admixture as the input variables. The variables of the neural network, in conjunction with the slurry density, viscosity, initial setting time, final setting time of each group of slurries, compressive strength and rate of stone formation of the formed stone body, serve as the output variables. The water-to-binder ratio refers to the ratio of the mass of water to the total mass of cementitious materials. The fly ash content by mass refers to the percentage of fly ash mass relative to the total mass of cementitious materials. The water glass volumetric dosage refers to the percentage of the volume of water glass relative to the total volume of the slurry. The reason for using volumetric measurement is that water glass is a viscous silicate solution; when preparing it in the laboratory or grouting at a construction site, measuring by volume is more convenient than weighing by mass. It also avoids errors caused by residue and material clinging to the sides of the container during the weighing of viscous liquids, and the volume can be converted to mass using the measured density. Slurry density refers to the ratio of the slurry’s density to that of water at the same temperature, indicating the slurry’s relative weight. Viscosity refers to the magnitude of internal frictional resistance when the slurry flows, reflecting its fluidity. Initial setting time refers to the time required from the start of mixing until the slurry begins to lose plasticity and undergoes initial setting. Final setting time refers to the time required from the start of mixing until the slurry completely loses plasticity and develops a certain strength. The compressive strength of the set mass refers to the maximum compressive stress that the set mass formed after the slurry has set can withstand under uniaxial compression. The stone rate refers to the ratio of the volume (V_1_) of the set mass formed after the slurry has set to the original volume (V_0_) of the slurry, reflecting the volume stability of the slurry after setting; it is calculated as V_1_/V_0_ × 100%.

**Figure 1:**
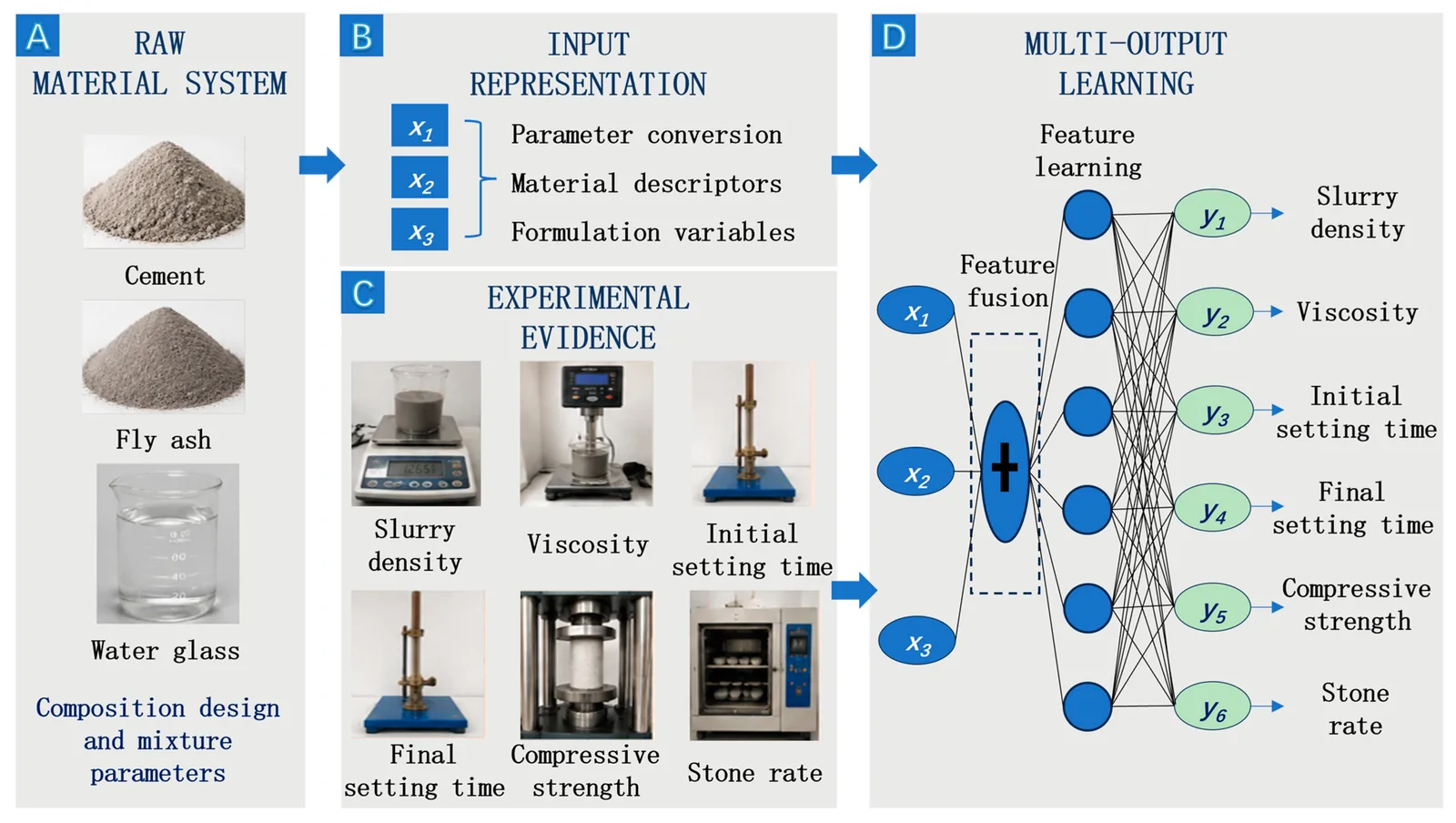
Diagram of raw materials and equipment.

**Figure 2:**
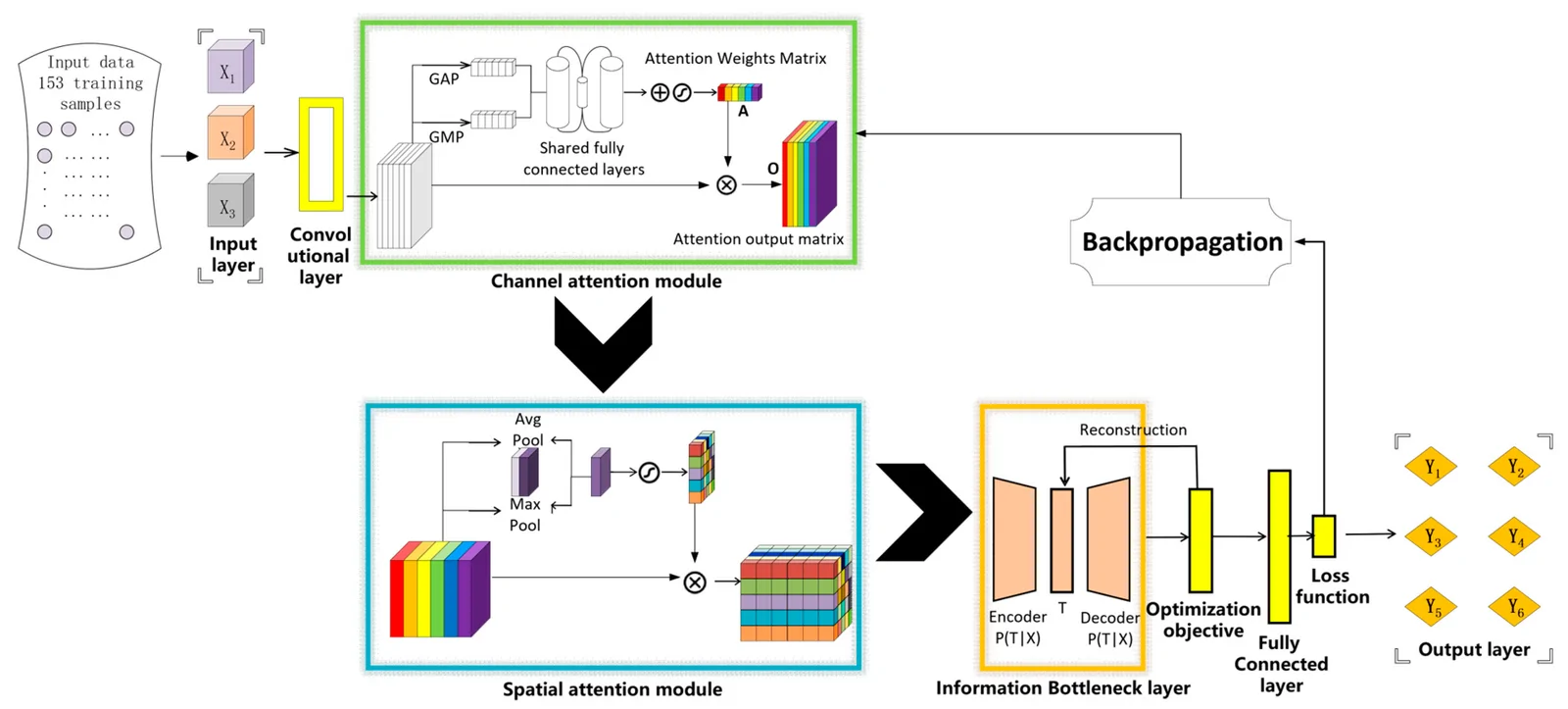
Architecture of neural network model.

Raw materials and testing instruments are illustrated in Figure 1. Employing convolutional neural networks in deep learning models facilitates enhanced adaptation to disparate data distributions, thereby rendering them more suitable for slurry performance prediction. The network model architecture is illustrated in Figure 2.

The raw materials used in this test included: Liquan Conch P·O 42.5 ordinary Portland cement (Liquan Conch Cement Co., Ltd., Xianyang, Shaanxi Province, China) (SiO_2_ 24.76%, CaO 57.81%, Al_2_O_3_ 6.19%), Gongyi Borun Grade II fly ash (Gongyi Borun Refractory Materials Co., Ltd., Gongyi, Henan Province, China) (16% retained on the 45 μm sieve, loss on ignition 2.8%, density 2.55 g/cm^3^; SiO_2_ 45.1%, Al_2_O_3_ 24.2%, CaO 5.6%), and Jiashan Yourui SP38 water glass solution (Jiashan Yourui Refractory Materials Co., Ltd., Jiashan County, Jiaxing, Zhejiang Province, China) (SiO_2_ 27.3%, Na_2_O 8.54%, density 38.5 Bé, modulus 3.30, solids content 35.84%). Regarding testing standards, raw materials were selected in accordance with GB/T 17671-2021 [30], YS/T 5211-2018 [31], SL/T 62-2020 [32], and JC/T 986-2018 [33]; the instruments used included an NDJ-8S rotary viscometer (Shanghai Yueping Scientific Instrument Co., Ltd., Shanghai, China), an NB-1 specific hydrometer (Shanghai Luda Experiment Instrument Co., Ltd., Shanghai, China), a mortar setting time tester (Hebei Tongli Instrument Equipment Co., Ltd., Cangzhou, China), a compression testing machine (Tianshui Hongshan Testing Machine Co., Ltd., Tianshui, China), and a micro-controlled electronic universal testing machine (Changchun Kexin Test Instrument Co., Ltd., Changchun, China). For specimen preparation, the modified slurry was prepared by dry-mixing cement and fly ash for 3 min, followed by wet mixing with water and water glass for 5 min. After molding, the mixture was compacted on a vibrating table, the mechanical test specimens were Φ50 mm × 100 mm cylinders, curing was conducted in a curing chamber, and the test age was 14 days.

The testing methods for each parameter are as follows: slurry density is determined directly using an NB-1 slurry density meter based on the lever balance principle; viscosity is calculated using an NDJ-8S rotational viscometer based on the Newtonian fluid model, converted from rotor torque; initial and final setting times are determined using a mortar setting time tester via the penetration resistance method, with penetration resistances of 0.3 MPa and 0.7 MPa, respectively, serving as the critical thresholds; compressive strength is calculated by dividing the failure load by the compressed area after loading the specimen to failure using a compression testing machine; and the stone rate is determined using the graduated cylinder method and is expressed as the percentage ratio of the volume of the solidified stone mass to the initial volume of the slurry after solidification.

### 2.1. Slurry Performance Prediction Model Based on CNN

Convolutional neural network (CNN) have been widely used for nonlinear feature extraction and prediction tasks. In this study, a CNN model was established to describe the nonlinear relationship between slurry mix proportions and performance indicators. The input variables consisted of three mix proportion parameters, including water-to-binder ratio, fly ash dosage, and water glass volumetric dosage. The output layer simultaneously predicted six performance indicators, including slurry density, viscosity, initial setting time, final setting time, compressive strength, and stone rate.

Although only three tabular variables were used as inputs, the relationship between slurry composition and performance is highly nonlinear because slurry properties are affected by the coupled effects of hydration reactions, particle packing, and chemical activation. Therefore, CNN was adopted as a nonlinear feature extraction framework rather than an image-processing model. The convolution operation was used to extract implicit interaction features among the mix proportion parameters, and the extracted features were subsequently mapped to multiple performance indicators through fully connected layers.

The basic structure of the CNN model consisted of an input layer, convolutional layer, activation function, pooling layer, fully connected layer, and output layer. The detailed architecture and parameters of the network are described in the following sections.

### 2.2. Attention Mechanism and Information Bottleneck Optimization

In order to improve the feature extraction capability of the CNN model, an attention mechanism was introduced to assign adaptive weights to different feature representations. The attention mechanism enables the model to focus on the input features that contribute more significantly to slurry performance prediction, thereby improving the representation ability of the network.

In this study, the attention mechanism was integrated into the CNN framework to enhance the extraction of nonlinear relationships between slurry mix proportions and performance indicators. The water-to-binder ratio, fly ash dosage, and water glass volumetric dosage may have different contributions to different performance indicators. Therefore, assigning adaptive weights to the extracted features can help the model capture the complex interactions among multiple input variables and output properties.

However, excessive retention of input information may introduce redundant features and increase the risk of overfitting, especially when the available experimental data are relatively limited. Therefore, information bottleneck theory was introduced to optimize the intermediate feature representation.

### 2.3. Information Bottleneck Theory Optimizes Slurry Prediction Model

Slonim, N and Tishby, N [34] were the first to propose the theory of information bottlenecks and proposed a new distributed clustering algorithm for explicitly maximizing the mutual information of the data and given categories between each cluster. Tishby, N., and Zaslavsky, N [35] employed the information bottleneck theory framework to model deep neural networks (DNNs), demonstrating that the information bottleneck theory can compute the optimal information-theoretic limit of DNNs and obtain finite sample generalization bounds. N Schwartz-Ziv and Tishby [36] presented a visual representation of the layers in the information plane, elucidating numerous intricacies of the internal mechanisms of deep learning and deep neural networks. Their findings demonstrated that DNN deep learning can be an effective approach for identifying an efficient representation of the minimum sufficient statistic in the theory of approximate information bottlenecks.

The research conducted by scholars on the information bottleneck theory demonstrates the viability of integrating this theory into deep learning, both in theoretical and experimental contexts. This paper introduces the information bottleneck theory into the domain of convolutional neural networks, with the objective of predicting performance to enhance the compactness and discriminativeness of the model representation by constraining the capacity of the intermediate representation, thereby improving the model’s robustness to noise and disturbances. This is particularly relevant in the context of fly ash slurry and the performance of the nodule body.

#### 2.3.1. Optimization Process

(1) Information bottleneck objective function

The information bottleneck theory aims to obtain an optimized intermediate representation by reducing redundant information from the input variables while preserving information related to the output variables. The optimization objective is expressed as:

(1) $$L_{IB}=I(X;T)-\beta I(T;Y)$$

(2) $$\underset{\theta }{\min}L_{IB}=I(X;T)-\beta I(T;Y)$$

where X represents the input variables, **T** represents the intermediate feature representation, **Y** represents the output performance indicators, and β is the balancing parameter controlling the trade-off between information compression and output information retention.

I(X;T) is the mutual information between the independent variable X and the intermediate representation T; I(T;Y) is the mutual information between the intermediate representation T and the dependent variable Y; and β is the equilibrium parameter.

(2) Definition of mutual information

Definition of mutual information $I\left(A;B\right)$:

(3) $$I(A;B)=\iint p(a,b)\log\frac{p(a,b)}{p(a)p(b)}dadb$$

For I(X;T) and I(T;Y), we respectively have:

(4) $$I(X;T)=\iint p(X,T)\log\frac{p(\left.T\right|X)}{p(T)}dTdX$$

(5) $$I(T;Y)=\iint p(T,Y)\log\frac{p(\left.Y\right|T)}{p(Y)}dTdY$$

Mutual information I(X;T) measures the degree of information association between the intermediate representation T and the input independent variable X; mutual information I(T;Y) measures the degree of information association between the intermediate representation T and the input independent variable Y.

(3) Approximate computation of mutual information

As the direct computation of mutual information is more complex, this paper uses variational approximation to facilitate the calculation.

Mutual information I(X;T) is approximated:

(6) $$I(X;T)\approx E_{p(X,T)}\left[\log\frac{q(\left.T\right|X)}{p(T)}\right]$$

Expand the independent variable matrix X in detail:

(7) $$X=\left[\begin{array}{ccc} x_{1}^{\left(1\right)} & x_{2}^{\left(1\right)} & x_{3}^{\left(1\right)}\\ x_{1}^{\left(2\right)} & x_{2}^{\left(2\right)} & x_{3}^{\left(2\right)}\\ \vdots & \vdots & \vdots \\ x_{1}^{\left(N\right)} & x_{2}^{\left(N\right)} & x_{3}^{\left(N\right)} \end{array}\right]$$

The middle representation matrix T is specified as:

(8) $$T=\left[\begin{array}{cccc} t_{1}^{\left(1\right)} & t_{2}^{\left(1\right)} & \cdots & t_{m}^{\left(1\right)}\\ t_{1}^{\left(2\right)} & t_{2}^{\left(2\right)} & \cdots & t_{m}^{\left(2\right)}\\ \vdots & \vdots & \ddots & \vdots \\ t_{1}^{\left(N\right)} & t_{2}^{\left(N\right)} & \cdots & t_{m}^{\left(N\right)} \end{array}\right]$$

The mutual information formula can be extended as:

(9) $$I\left(X;T\right)=\sum_{i=1}^{N}\sum_{j=1}^{m}E\left[\log\frac{p\left(\left.t_{j}^{\left(i\right)}\right|x_{1}^{\left(i\right)},x_{2}^{\left(i\right)},x_{3}^{\left(i\right)}\right)}{p\left(t_{j}^{\left(i\right)}\right)}\right]$$

Mutual information $I(T;Y)\sqrt{a^{2}+b^{2}}$ is approximated:

(10) $$I(T;Y)\approx E_{p(T,Y)}\left[\log\frac{q(\left.Y\right|T)}{p(Y)}\right]$$

Expand the dependent variable matrix Y in detail:

(11) $$Y=\left[\begin{array}{cccccc} y_{1}^{\left(1\right)} & y_{2}^{\left(1\right)} & y_{3}^{\left(1\right)} & y_{4}^{\left(1\right)} & y_{5}^{\left(1\right)} & y_{6}^{\left(1\right)}\\ y_{1}^{\left(2\right)} & y_{2}^{\left(2\right)} & y_{3}^{\left(2\right)} & y_{4}^{\left(2\right)} & y_{5}^{\left(2\right)} & y_{6}^{\left(2\right)}\\ \vdots & \vdots & \vdots & \vdots & \vdots & \vdots \\ y_{1}^{\left(N\right)} & y_{2}^{\left(N\right)} & y_{3}^{\left(N\right)} & y_{4}^{\left(N\right)} & y_{5}^{\left(N\right)} & y_{6}^{\left(N\right)} \end{array}\right]$$

The mutual information formula can be extended as:

(12) $$I\left(T;Y\right)=\sum_{i=1}^{N}\sum_{k=1}^{6}E\left[\log\frac{p\left(\left.y_{k}^{\left(i\right)}\right|t_{1}^{\left(i\right)},t_{2}^{\left(i\right)},\cdots ,t_{m}^{\left(i\right)}\right)}{p\left(y_{k}^{\left(i\right)}\right)}\right]$$

(4) Construction of the loss function

Combining the above approximations, the loss function of the information bottleneck can be expressed as:

(13) $$L_{IB}=E_{p(X,Y)}\left[\log q(\left.T\right|X)\right]-\beta E_{p(T,Y)}\left[\log q(\left.Y\right|T)\right]$$

Substitute the extended mutual information expression into the objective function as:

(14) $$L_{IB}=\sum_{i=1}^{N}\sum_{j=1}^{m}E\left[\log\frac{p\left(\left.t_{j}^{\left(i\right)}\right|x_{1}^{\left(i\right)},x_{2}^{\left(i\right)},x_{3}^{\left(i\right)}\right)}{p\left(t_{j}^{\left(i\right)}\right)}\right]-\beta \sum_{i=1}^{N}\sum_{k=1}^{6}E\left[\log\frac{p\left(\left.y_{k}^{\left(i\right)}\right|t_{1}^{\left(i\right)},t_{2}^{\left(i\right)},\cdots ,t_{m}^{\left(i\right)}\right)}{p\left(y_{k}^{\left(i\right)}\right)}\right]$$

This objective function clearly shows how the independent variable $x_{1},x_{2},x_{3}$ affects the final dependent variable $y_{1},y_{2},\cdots ,y_{6}$ through the intermediate representation $t_{1},t_{2},\cdots t_{m}$ and how the balance between input and output information is controlled during the optimization process.

(5) Final loss function

The final loss function of the integrated model is:

(15) $$L=L_{pred}+\lambda_{AM}L_{AM}+\lambda_{IB}L_{IB}$$

$L_{pred}$ is the prediction loss, $L_{AM}$ is the loss of the attention mechanism, $L_{IB}$ is the loss of the information bottleneck, and $\lambda_{AM}$ and $\lambda_{IB}$ are the corresponding equilibrium parameters.

#### 2.3.2. Model Advantage

In the actual training process, the parameter uptake is often unavoidable. The superposition and nesting of a single traditional neural network is difficult to constitute an effective theoretical proof and practical effectiveness. The information bottleneck theory optimized convolutional neural network (IB-CNN) has the following advantages over the ordinary convolutional neural network model:

(1) Reduction in redundant information and overfitting risk

By minimizing I(X;T), the information bottleneck reduces the redundant information in the input variable, i.e., slurry ratios, into the intermediate representation T and reduces the dependence of the intermediate representation T on the slurry ratios, thus removing the redundant information and noise in the input variable, reducing the risk of overfitting, and preventing the data from being overfitted due to the concentration of data in the input variable for the performance prediction:

(16) $$E_{p(X)}\left[D_{KL}(q(\left.T\right|X)\left\|p(T)\right.)\right]$$

(2) Improve generalization capabilities

By maximizing I(T;Y), the information bottleneck ensures that the intermediate representation T retains as much information relevant to the slurry performance Y as possible and thus improves the accuracy and generalization of the slurry performance prediction:

(17) $$E_{p(T)}\left[D_{KL}(q(\left.Y\right|T)\left\|p(Y)\right.)\right]$$

(3) Balancing information retention and compression

The information bottleneck is optimally balanced between prediction performance and model complexity by controlling the degree of information retention and compression through the balancing parameter β, which ensures discrete slurry ratios and comprehensive prediction of slurry performance:

(18) $$L=E_{p(X,Y)}\left[-\log q(\left.Y\right|T)\right]+\beta E_{p(X)}\left[D_{KL}(q(\left.T\right|X)\left\|p(T)\right.)\right]$$

### 2.4. Model Training and Validation Strategy

The data of a total of 153 datasets collected from our experiments were used for model training and validation. The input variables included water-to-binder ratio, fly ash dosage, and water glass volumetric dosage, while six output variables were considered, including slurry density, viscosity, initial setting time, final setting time, compressive strength, and stone rate.

Four models, including the original CNN model, information-bottleneck-optimized CNN (IB-CNN), attention-mechanism-optimized CNN (AM-CNN), and information-bottleneck–attention-optimized CNN (IB-AM-CNN), were established for comparison. All models were trained under identical conditions to ensure the reliability of the comparison results.

The datasets were randomly divided into training and validation sets with a ratio of 8:2. The Adam optimizer was adopted for model training, with an initial learning rate of 0.001. The batch size was set to 32, and the maximum training epochs was 500. To prevent overfitting caused by the limited dataset size, early stopping was applied. Training was terminated when the validation loss did not decrease for 20 consecutive iterations.

The final loss function of the proposed model consisted of three components: prediction loss, attention mechanism loss, and information bottleneck loss. The weighting parameters were set as α = 0.1 and β = 0.01, where α controls the contribution of the attention mechanism and β controls the balance between information compression and output information retention.

The prediction performance of different models was evaluated using the same validation dataset, and the prediction results were compared based on the relative errors between the predicted values and experimental measurements.

## 3. Results

To ascertain the efficacy of the model training, fly ash slurry proportioning and performance tests were conducted, and the test outcomes were employed as a validation set of non-master data, thereby ensuring the accuracy and reliability of the model validation.

### 3.1. Raw Materials and Test Groups

The raw materials utilized in the production of fly ash slurry include water, cement, fly ash, and water glass, among others. The cement selected is standard 425 ordinary silicate cement, while the fly ash is of the second level. Water glass, as a silicate solution, has the chemical formula Na_2_O · n SiO_2_. This can be used in two ways: firstly, as an exciter for the slurry to provide an alkaline environment; secondly, as a reactant to generate colloids.

The mix design was conducted using an orthogonal experimental design, treating the water-to-binder ratio, the mass fraction of fly ash, and the volumetric fraction of water glass as three factors. An L9(3^3^) orthogonal experiment was designed, incorporating the actual quantities of water, cement, fly ash, and water glass solution; the relevant groupings are shown in Table 1a. This experimental design was selected because, as an exploratory study, the L9(3^3^) orthogonal array requires only nine test groups to effectively evaluate the main effects of each of the three factors. It is highly efficient under conditions of limited experimental resources, and the three factors at three levels cover the actual range of fly ash grout mix proportions used in grouting projects. Although the L9 orthogonal array cannot completely isolate the interaction effects between two factors, range analysis (Table 1b) can reliably assess the relative importance of each factor on grout performance.

### 3.2. Results of the Experiment

According to the test grouping, the slurry density, viscosity, initial setting time, final setting time of each group of slurry were determined in a total of four performance indicators, and the compressive strength of the stone body and the stone rate of two performance indicators, which are shown in Table 2.

For each mix design, three replicate tests were conducted for each performance indicator. The experimental results are expressed as the mean ± standard deviation (SD, *n* = 3). Both the mean and standard deviation were calculated directly from the three individual replicate measurements. No assumed coefficient of variation was used to estimate the standard deviation. The measured slurry density, viscosity, initial setting time, final setting time, compressive strength, and stone rate are summarized in Table 2.

The optimized model combines the feature extraction capability of convolutional neural networks, the feature selection capability of attention mechanisms and the optimized capability of information bottleneck theory for the prediction of Output Layer $\hat{Y}$. The theoretical performance of the optimization has been demonstrated in the previous chapter, and the training of the model is carried out in this chapter.

### 3.3. Training of the Model

A total of 153 sets of existing data were collated from our experiments and collected as training samples. To improve the traceability of the dataset, each data record was assigned a unique identification number, and the corresponding source information was provided in the supplementary dataset. These samples were selected based on three input variables, X, namely water-to-binder ratio, fly ash dosage and water glass volumetric dosage. The output variables, Y, which were used to assess the performance of the samples, included slurry density, viscosity, initial setting time, final setting time, compressive strength and stone rate. The training process employed the use of four distinct types of convolutional neural networks (CNNs), namely: (1) CNNs optimized using information bottleneck theory (IB-CNN), (2) CNNs optimized using attention theory (AM-CNN), (3) CNN models optimized using information bottleneck theory coupled with attention theory (IB-AM-CNN), and (4) CNNs that were not subjected to any optimization process (non-optimized CNNs).

Before model training, the collected datasets were screened according to unified criteria. The selected datasets satisfied the following requirements: (1) the slurry system consisted of cement, fly ash, water, and water glass; (2) complete information of mix proportions and performance indicators was available; and (3) the experimental results could be converted into the six target performance indicators considered in this study.

Since the datasets were obtained from different published studies, differences may exist in raw materials, mixture proportion definitions, testing methods, specimen dimensions, curing ages, and curing conditions. To improve data comparability, the collected data were carefully screened before model training.

**Figure 3:**
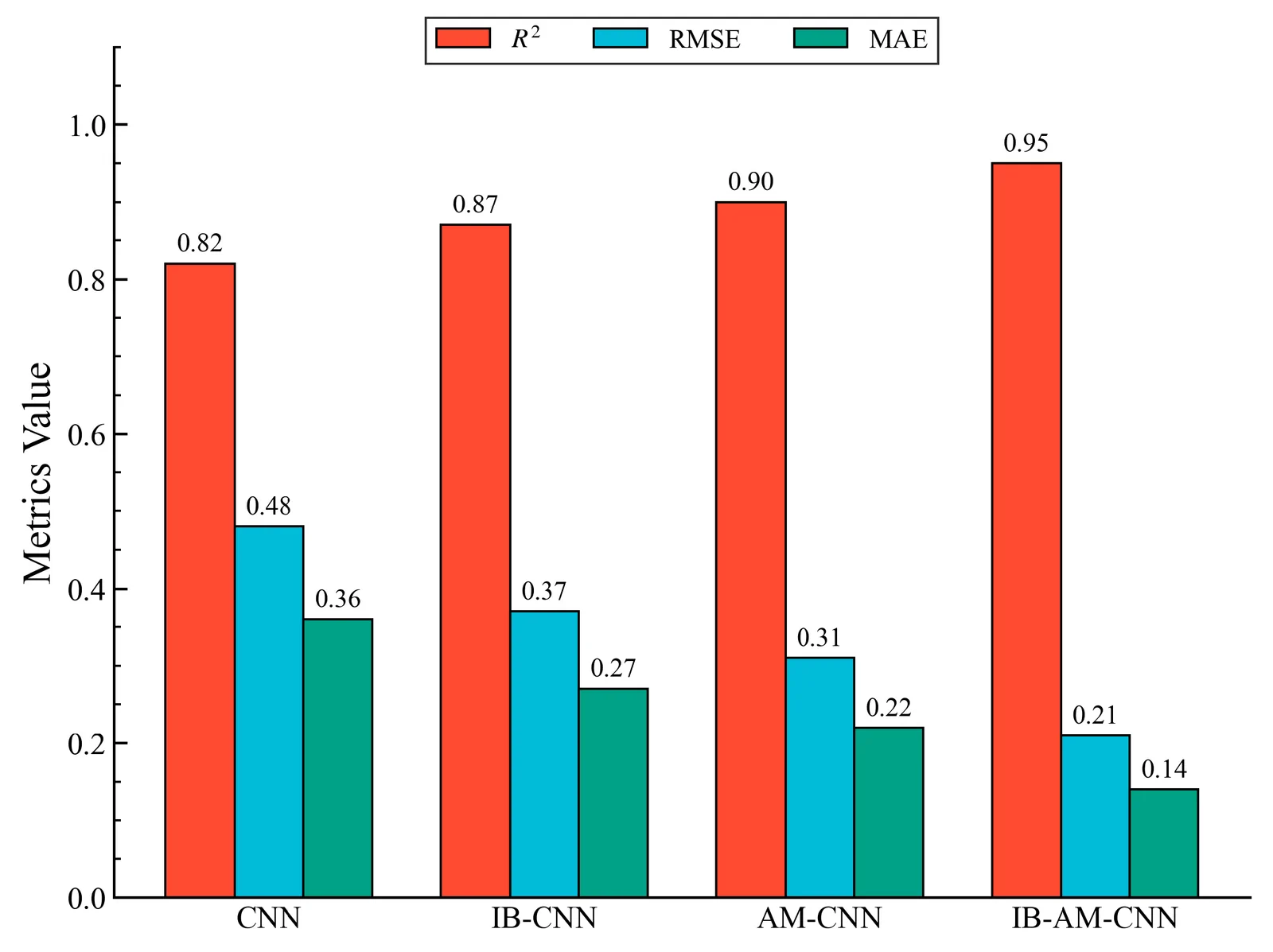
Overall performance comparison of different models based on five-fold cross-validation in the normalized output space.

Before training the prediction models, all input and output variables were normalized to eliminate the influence of different numerical ranges and units. During five-fold cross-validation, the performance metrics shown in Figure 3 were calculated in the normalized output space. For each fold, R^2^, RMSE, and MAE were first calculated separately for each of the six normalized output variables, and the overall values shown in Figure 3 were obtained by averaging the corresponding metrics across the six outputs and five validation folds. Therefore, the RMSE and MAE values shown in Figure 3 are dimensionless normalized-scale metrics and are intended for overall comparison among the four models.

To provide physically interpretable prediction errors, the predicted and measured values were subsequently transformed back to their original physical units. R^2^, RMSE, and MAE were then reported separately for each output variable, as summarized in Table 3.

The model was trained using the Adam optimizer, with an initial learning rate of 0.001, a batch size of 32, and a maximum of 500 training iterations. The 153 datasets were randomly split into a training set and a validation set in an 8:2 ratio, and an early stopping strategy was employed: training was terminated prematurely when the validation set loss failed to decrease for 20 consecutive iterations to prevent overfitting. The model’s loss function consists of three components: prediction loss, attention mechanism loss, and information bottleneck loss. The corresponding balancing parameters are set to α = 0.1 and β = 0.01, where β is used to control the trade-off between input information compression and output information retention. The four models—CNN, IB-CNN, AM-CNN, and IB-AM-CNN—were all trained under exactly the same training parameter settings to ensure the fairness and reliability of the comparative results. This study employs a single six-output model—that is, a convolutional neural network (CNN) that simultaneously outputs six performance metrics, rather than six independent models.

To reduce the influence of random data partitioning on model evaluation, five-fold cross-validation was additionally performed during model training. The complete dataset was randomly divided into five subsets, with four subsets used for training and the remaining subset used for validation. This process was repeated five times, and the average performance obtained from the five validation results was used to evaluate the robustness of different models.

In each fold, model performance was evaluated separately for the six output variables. The normalized-space metrics were used for the overall comparison in Figure 3, whereas the inverse-transformed results in the original physical units were used for the output-specific evaluation in Table 3.

R^2^, RMSE, and MAE represent the mean values across the six output variables and five validation folds. RMSE and MAE in this figure are dimensionless because they were calculated using normalized outputs.

Values in each cell are presented as R^2^/RMSE/MAE. R^2^ is dimensionless. RMSE and MAE were calculated after inverse transformation of the normalized predictions and are therefore expressed in the original unit of each corresponding output variable. The reported values represent the mean performance over the five validation folds.

As shown in Figure 3, the five-fold cross-validation results in the normalized output space indicate that the IB-AM-CNN model achieved the best overall performance among the four models, with the highest mean coefficient of determination (R^2^ = 0.95) and the lowest normalized RMSE and MAE values of 0.21 and 0.14, respectively. The corresponding R^2^ values of the CNN, IB-CNN, and AM-CNN models were 0.82, 0.87, and 0.90, respectively.

Because the six target variables have substantially different physical scales and units, the normalized aggregate RMSE and MAE values in Figure 3 are primarily used for overall model comparison and should not be interpreted as errors in physical units. Therefore, the prediction results were inverse-transformed to the original units, and R^2^, RMSE, and MAE were calculated separately for each target variable, as shown in Table 3.

The output-specific results further show that the IB-AM-CNN model consistently achieved higher R^2^ values and lower RMSE and MAE values than the other three models for all six performance indicators. For the IB-AM-CNN model, the R^2^ values ranged from 0.94 to 0.96. The corresponding RMSE values were 0.043 g/cm^3^ for density, 4.60 Pa·s for viscosity, 88 min for initial setting time, 132 min for final setting time, 0.45 MPa for compressive strength, and 3.40% for stone rate. These results provide a more physically interpretable evaluation of the prediction performance for each slurry property.

### 3.4. Validation of Prediction Accuracy

For a total of six parameters, namely, slurry density, viscosity, initial setting time, final setting time, compressive strength, and stone rate, the corresponding CNN model, IB-CNN model, and AM-CNN model were trained for each parameter, and the prediction results of the CNN model were taken as an example:

(19) $$\hat{Y}=\left[\begin{array}{cccccc} y_{1}^{\left(1\right)} & y_{2}^{\left(1\right)} & y_{3}^{\left(1\right)} & y_{4}^{\left(1\right)} & y_{5}^{\left(1\right)} & y_{6}^{\left(1\right)}\\ y_{1}^{\left(2\right)} & y_{2}^{\left(2\right)} & y_{3}^{\left(2\right)} & y_{4}^{\left(2\right)} & y_{5}^{\left(2\right)} & y_{6}^{\left(2\right)}\\ \vdots & \vdots & \vdots & \vdots & \vdots & \vdots \\ y_{1}^{\left(N\right)} & y_{2}^{\left(N\right)} & y_{3}^{\left(N\right)} & y_{4}^{\left(N\right)} & y_{5}^{\left(N\right)} & y_{6}^{\left(N\right)} \end{array}\right]=\left[\begin{array}{llllll} 1.32 & 21.09 & 997.82 & 2069.1 & 1.22 & 69.51\\ 1.95 & 33.42 & 1519.75 & 2417.23 & 3.48 & 81.51\\ 1.94 & 34.27 & 1614.52 & 1791.52 & 3.42 & 74.53\\ 1.27 & 39.31 & 1091.97 & 1748.36 & 4.89 & 79.78\\ 1.79 & 16.21 & 1191.52 & 1952.12 & 4.53 & 72.59\\ 1.23 & 15.45 & 1810.12 & 3100.62 & 4.18 & 91.51\\ 1.18 & 20.31 & 1724.51 & 2614.71 & 5.97 & 89.63\\ 1.7 & 12.93 & 1241.52 & 2710.19 & 7.55 & 84.6\\ 1.77 & 52.96 & 1382.54 & 3312.89 & 4.74 & 63.72 \end{array}\right]$$

**Figure 4:**
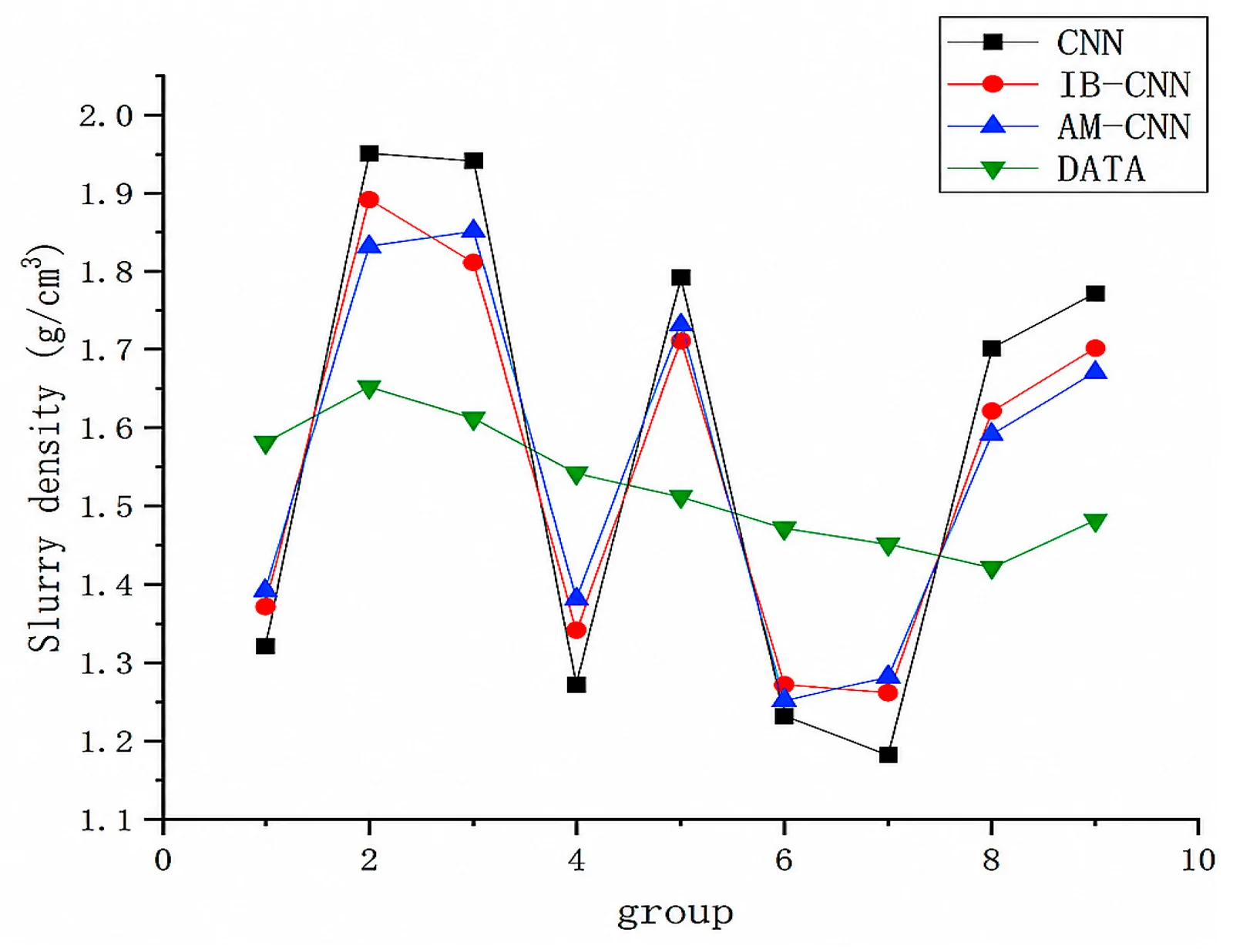
Prediction results of slurry density.

**Figure 5:**
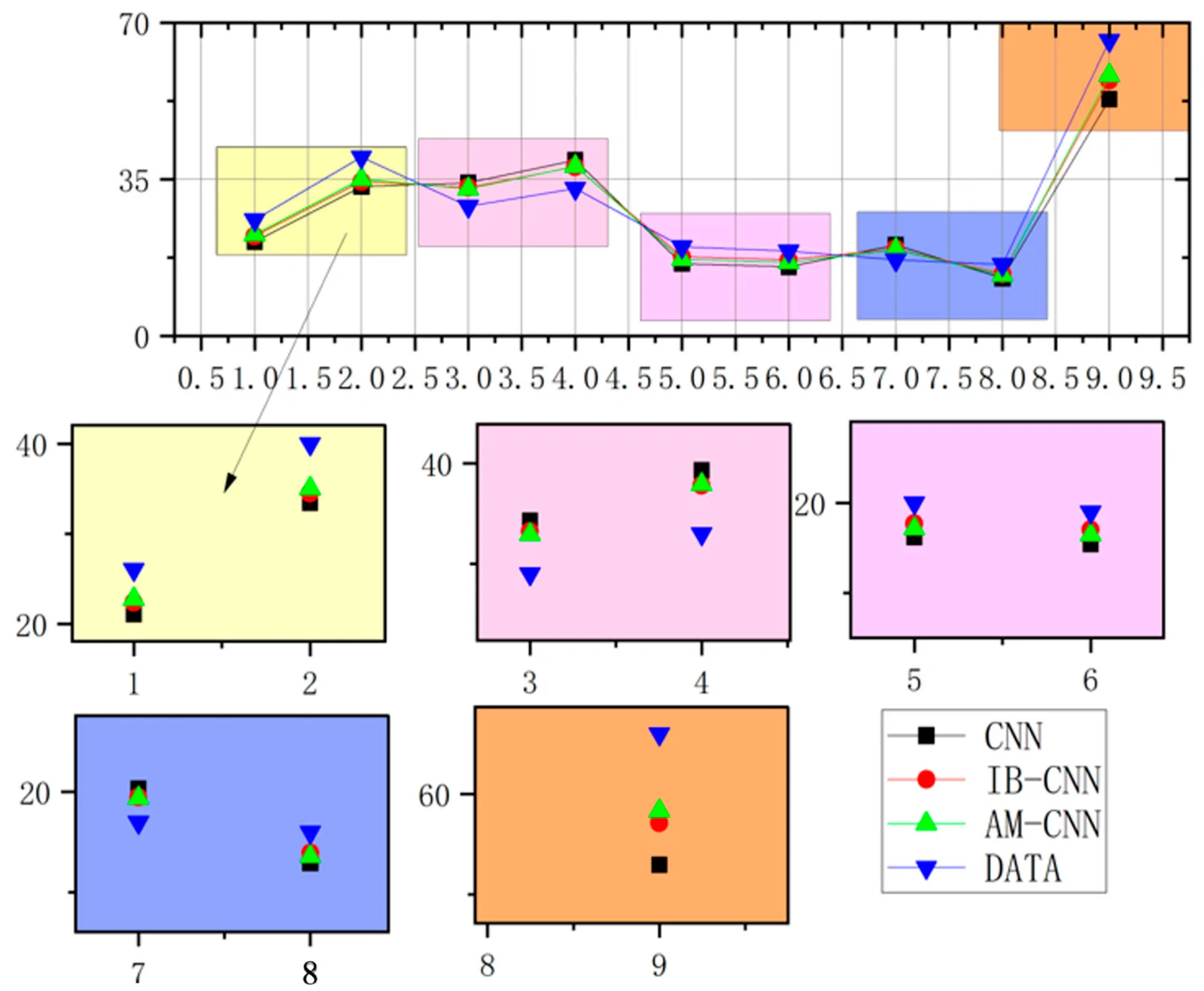
Prediction results of viscosity.

**Figure 6:**
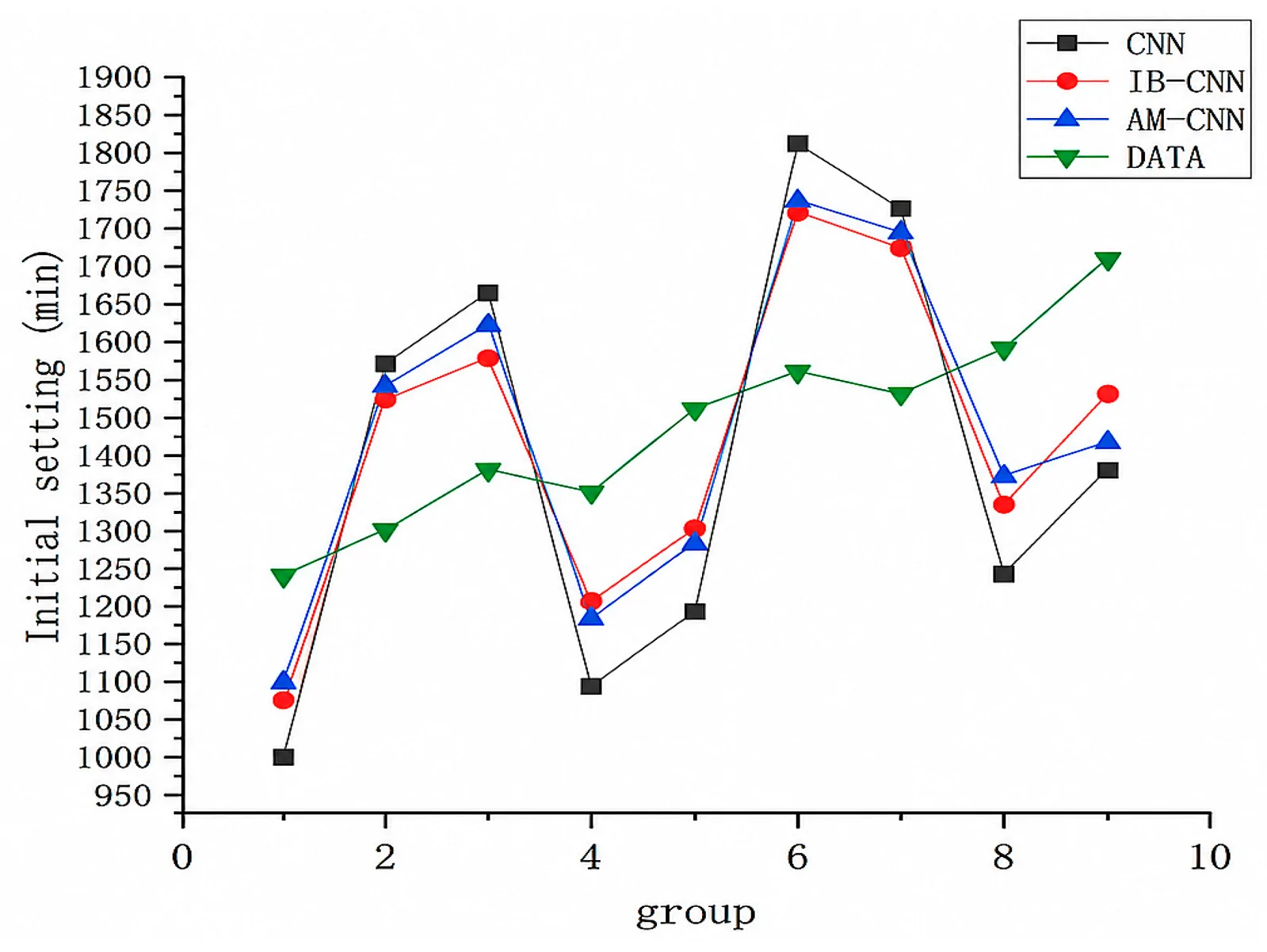
Prediction results of initial setting time.

**Figure 7:**
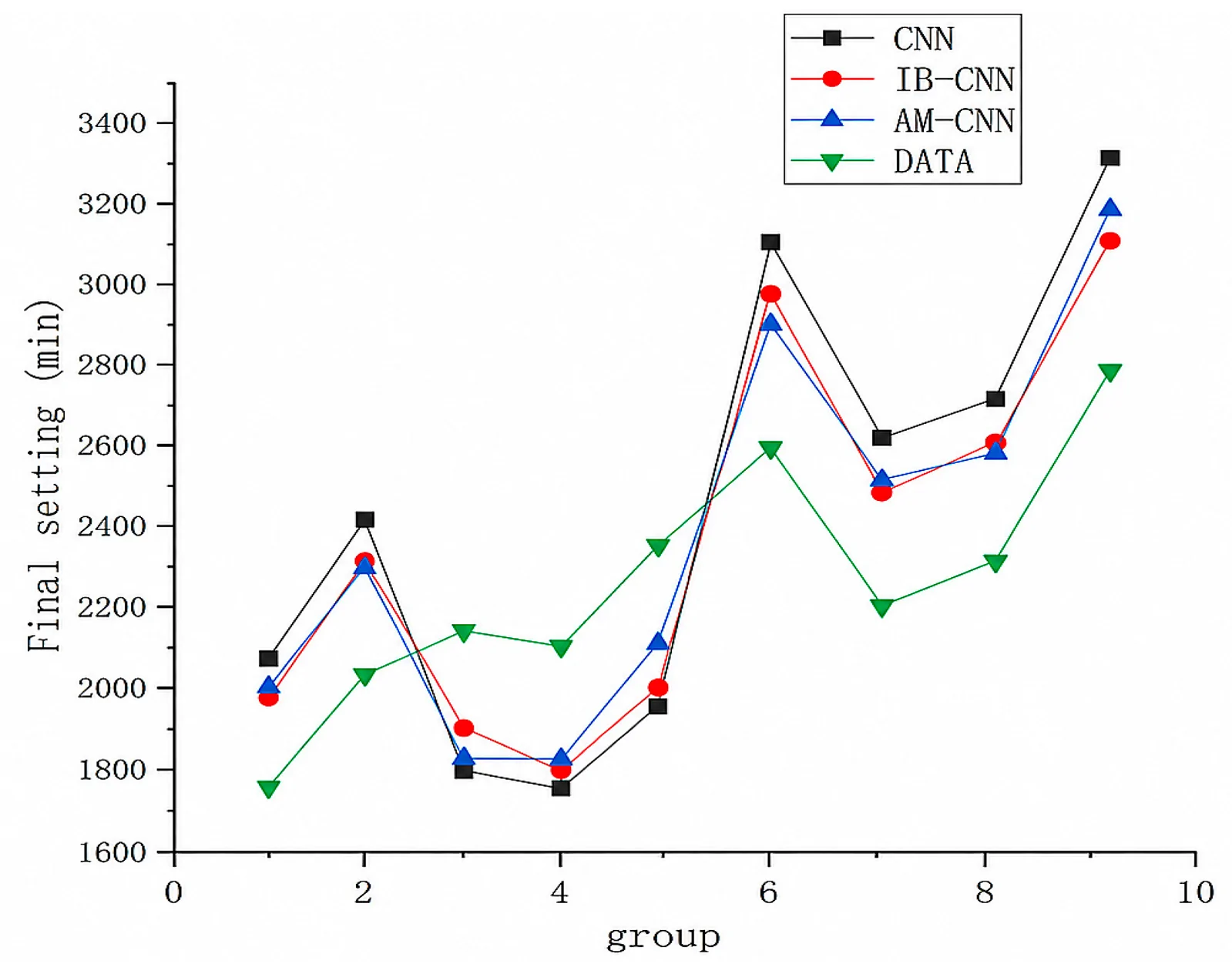
Prediction results of final setting time.

**Figure 8:**
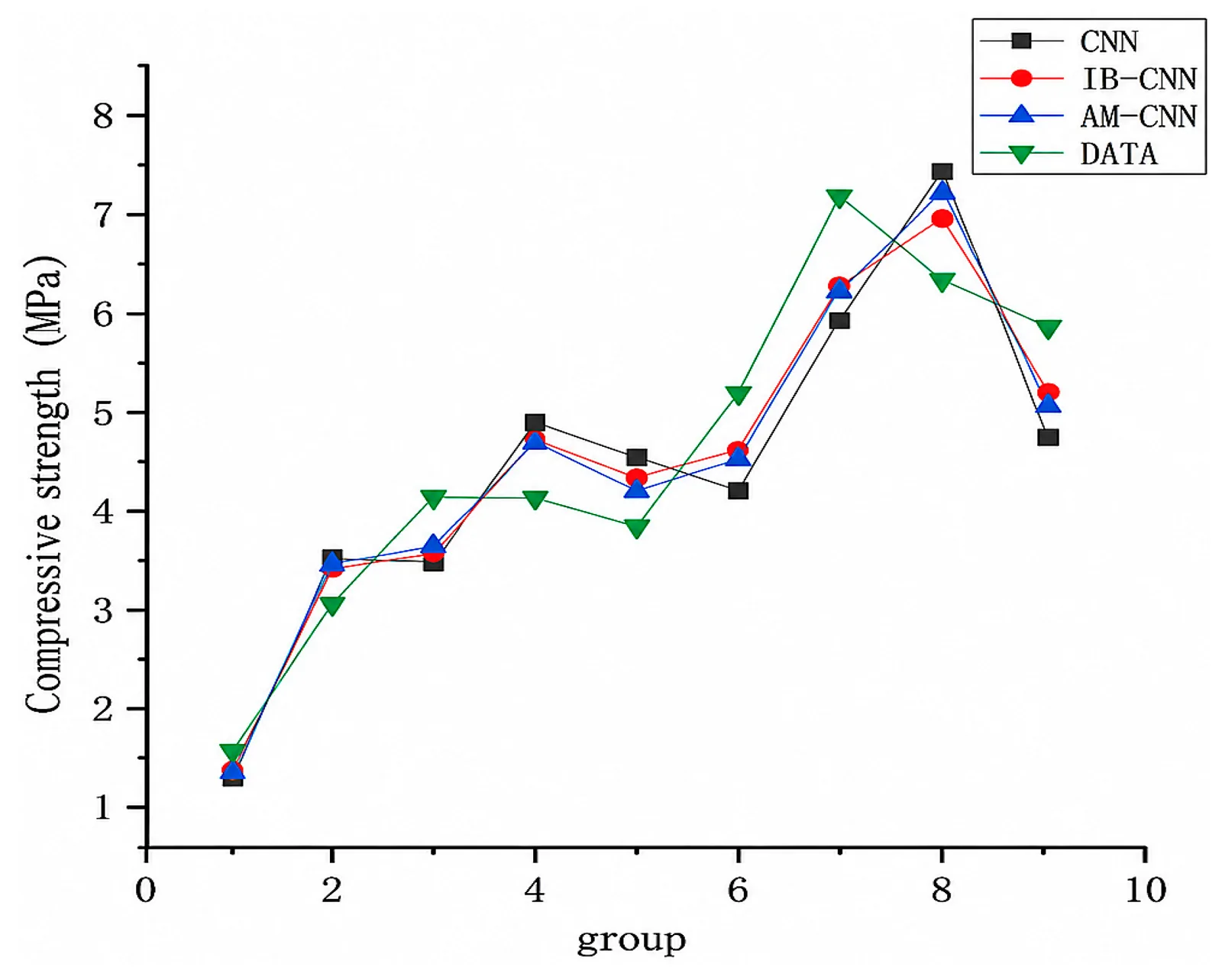
Prediction results of compressive strength.

**Figure 9:**
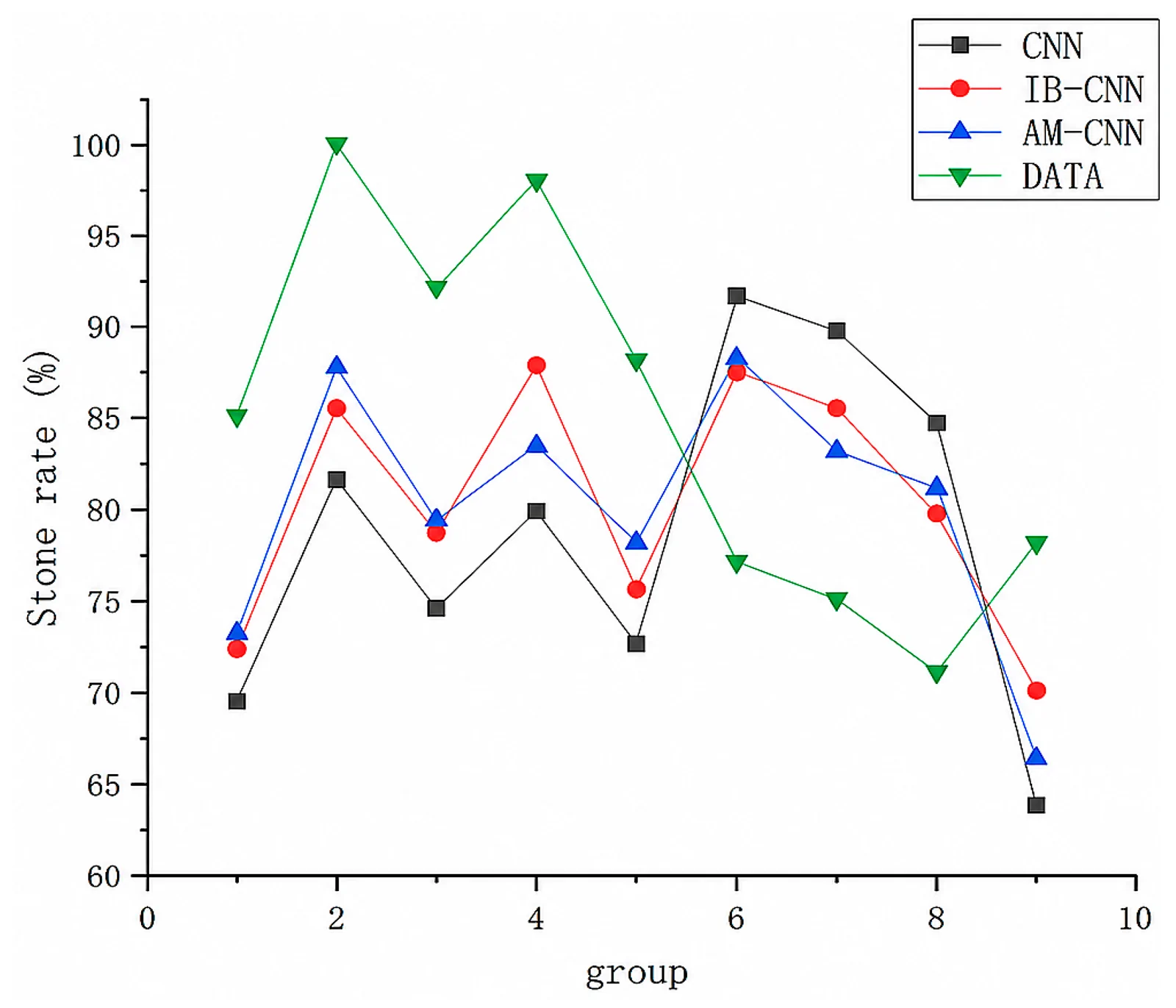
Prediction results of stone rate.

The results of predicting each parameter of each model are shown in Figure 4, Figure 5, Figure 6, Figure 7, Figure 8 and Figure 9, where the CNN model, IB-CNN model, and AM-CNN model are the prediction results ${\hat{y}}^{\left(i\right)}$ of each model, which are compared with the non-master DATA group.

Figure 5 illustrates that the prediction results of viscosity are more accurate and precise than the other parameters, which is due to the fact that viscosity characterizes the magnitude of the viscosity of the slurry. The viscosity is less affected by the input variables (water-to-binder ratio, fly ash dosage, and water glass volumetric dosage) than the other coefficients are affected by the input variables. The model it trains has a better robustness. For the model prediction of other parameters, the degree of deviation between groups for each parameter is larger, which is caused by the irrelevance of the input variables of each group in the orthogonal experimental design (Table 1a), but does not affect the accuracy of the prediction model. As can be seen from Figure 4, Figure 5, Figure 6, Figure 7, Figure 8 and Figure 9, the data of IB-CNN and AM-CNN models are closer to the real data (DATA) obtained from the experiment than the CNN model, which has a smaller degree of deviation from the real data obtained from the experiment (DATA).

**Figure 10:**
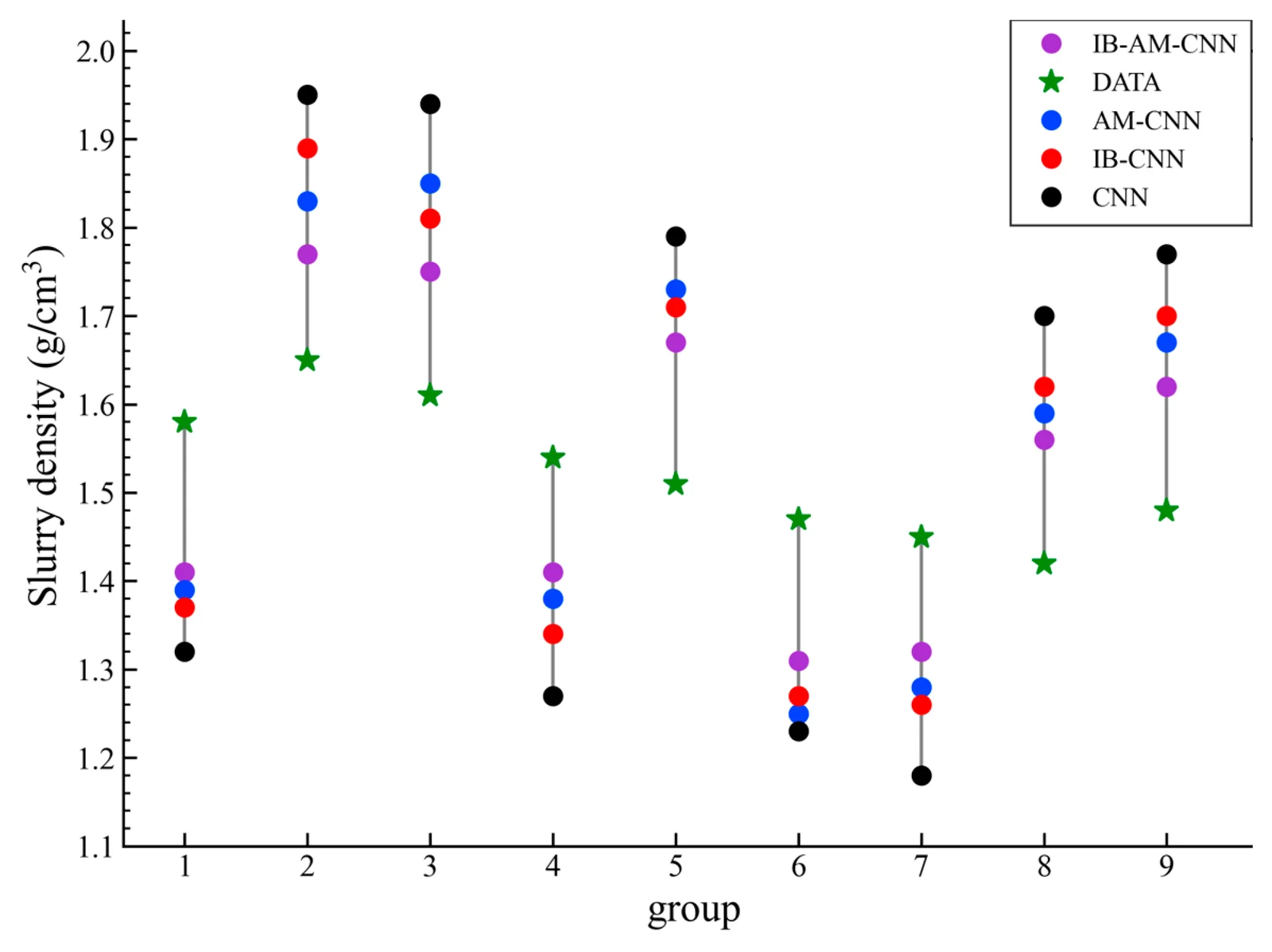
Comparison of measured and predicted slurry density for the nine experimental groups.

**Figure 11:**
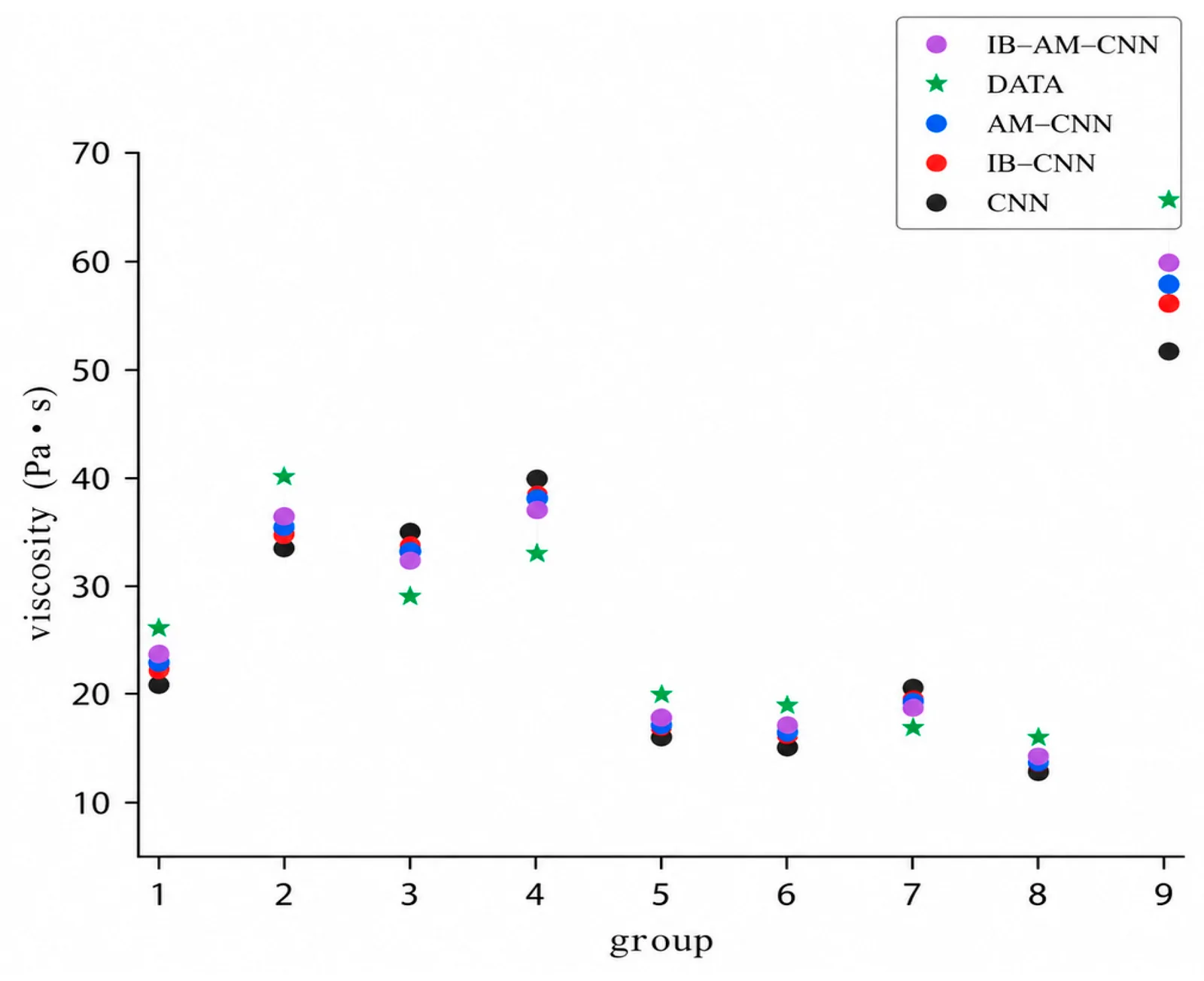
Comparison of measured and predicted slurry viscosity for the nine experimental groups.

**Figure 12:**
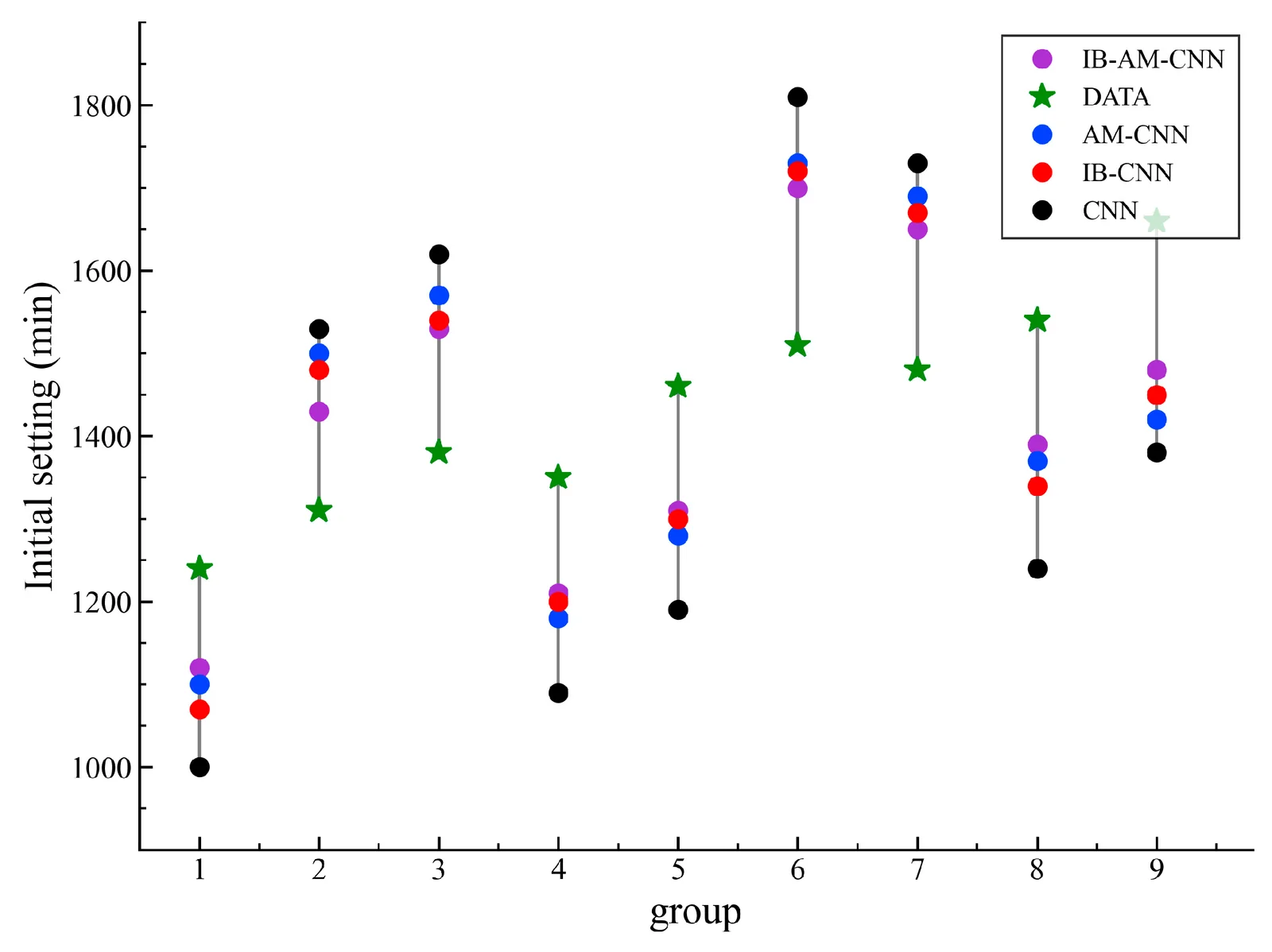
Comparison of measured and predicted initial setting time for the nine experimental groups.

**Figure 13:**
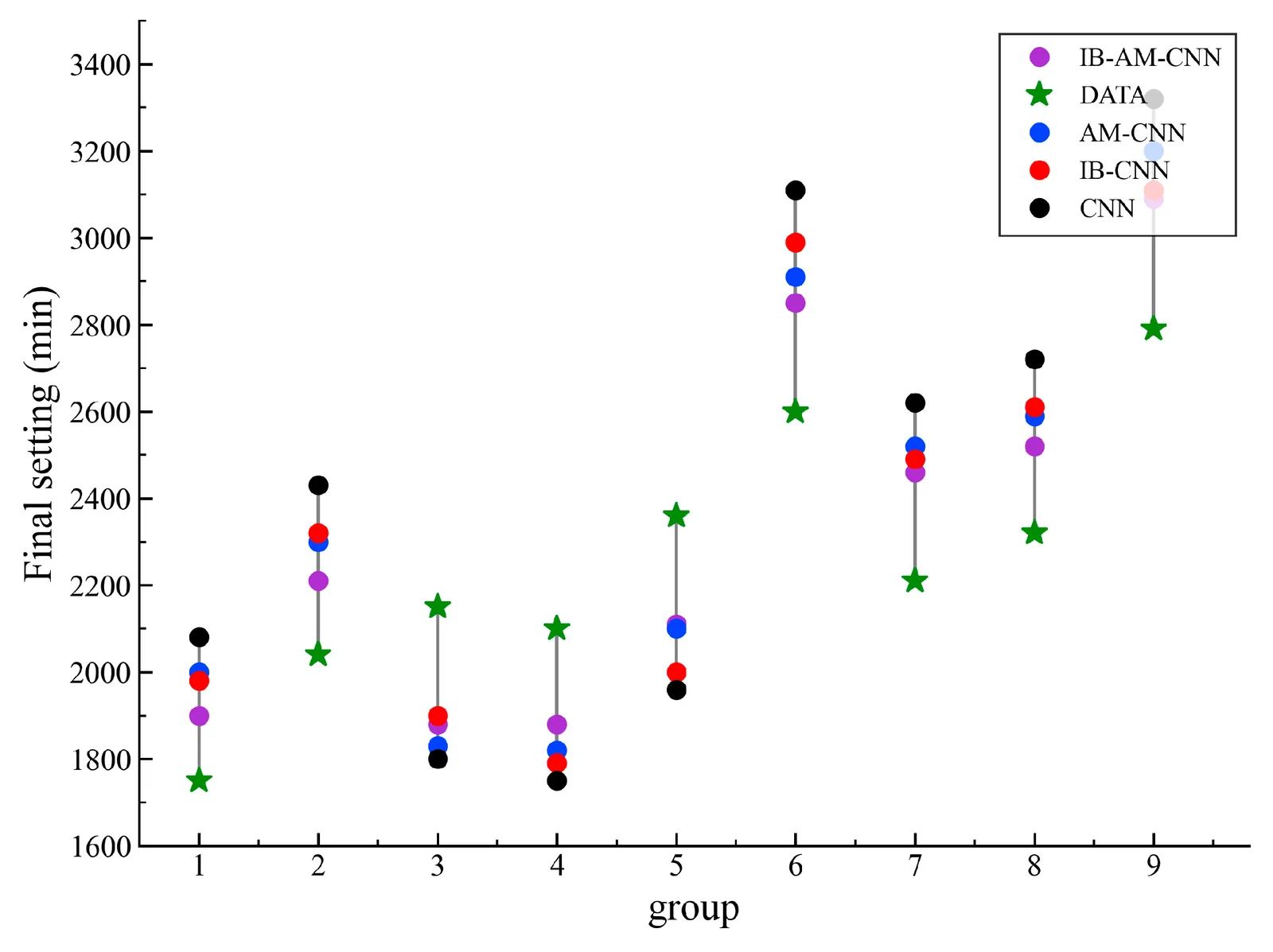
Comparison of measured and predicted final setting time for the nine experimental groups.

**Figure 14:**
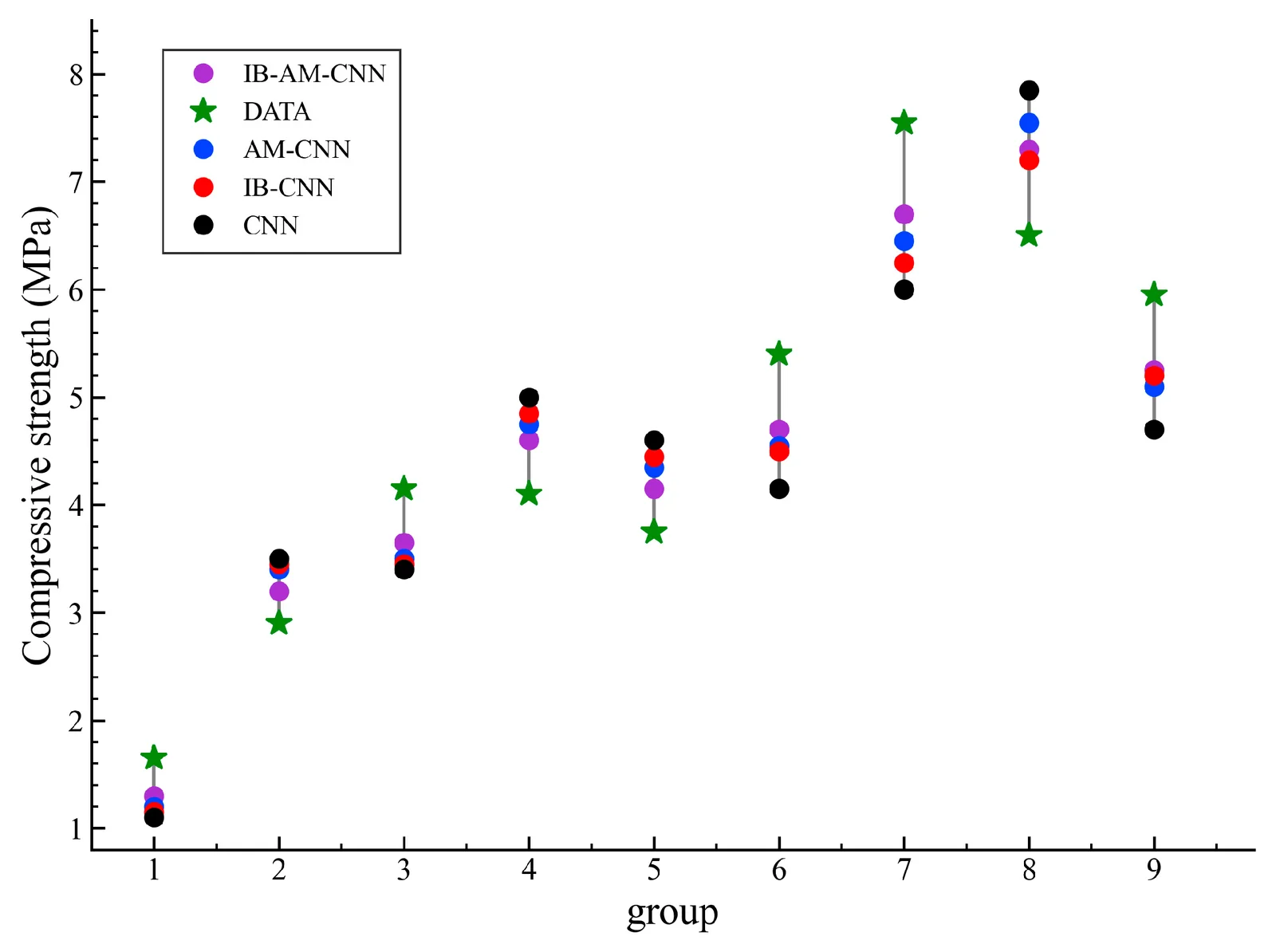
Comparison of measured and predicted compressive strength for the nine experimental groups.

**Figure 15:**
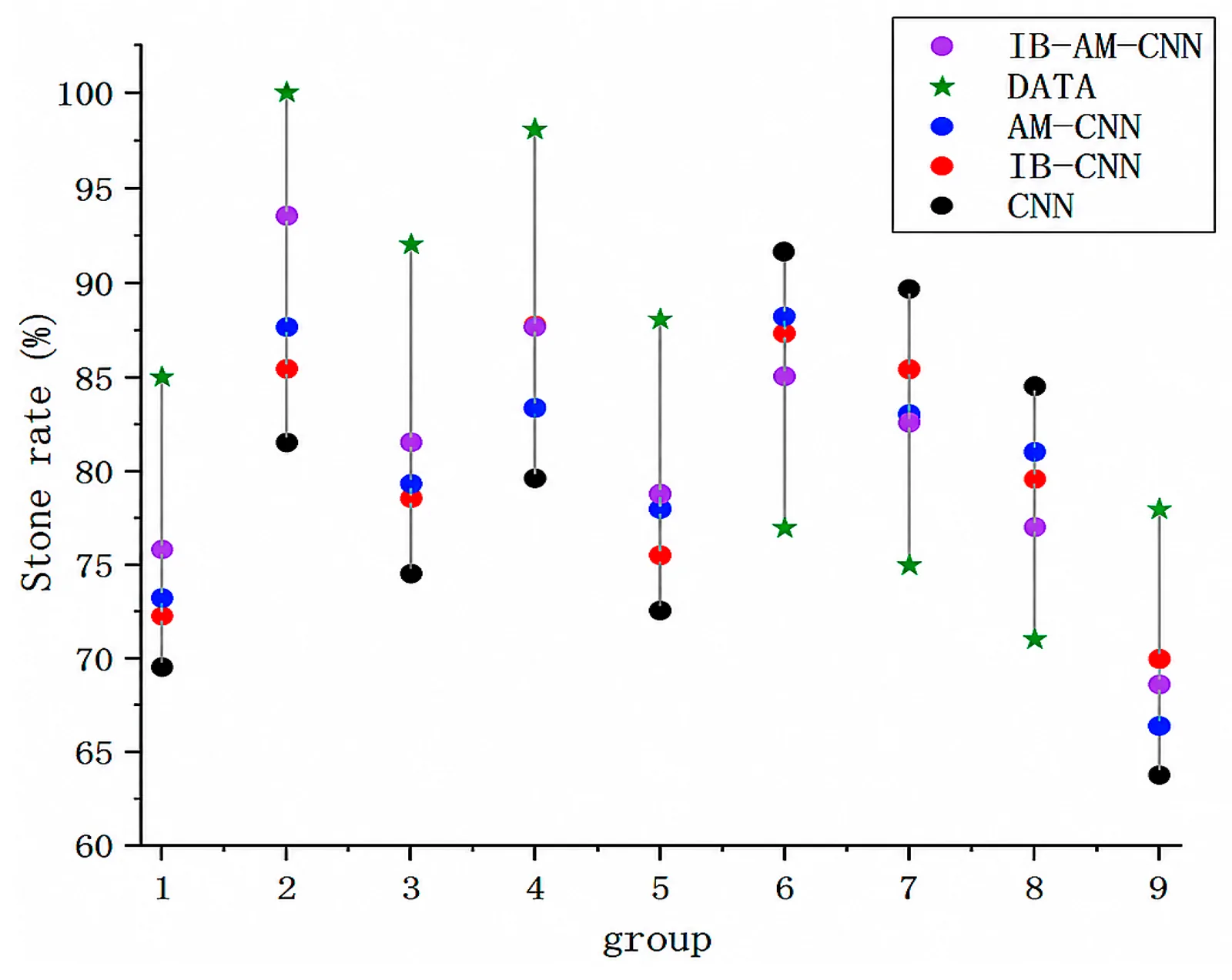
Comparison of measured and predicted stone rate for the nine experimental groups.

To further compare the predictive performance of the four models, Figure 10, Figure 11, Figure 12, Figure 13, Figure 14 and Figure 15 present the experimentally measured values (DATA) together with the predictions obtained using CNN, IB-CNN, AM-CNN, and IB-AM-CNN for the nine experimental groups. Each marker represents the measured or predicted value of an individual experimental group. The figures are intended to provide a direct visual comparison of the deviations between the model predictions and the experimental measurements. To avoid statistical misinterpretation, the shaded trend bands shown in the previous version have been removed because they were used only as visual guides and did not represent statistically derived confidence or prediction intervals.

As can be seen from Figure 10, Figure 11, Figure 12, Figure 13, Figure 14 and Figure 15, the groups predicted by the IB-AM-CNN model are closer to the DATA group data of the real test than the CNN, IB-CNN and AM-CNN, the prediction accuracy of each parameter has been optimized, and the predicted data show that both information bottleneck and attention theories have a role in improving the prediction accuracy of the convolutional neural network model for prediction of the performance of the slurry.

Take the prediction results of each prediction model and compare them with the real data to determine the relative error of each group of prediction models, take the average of the relative error of each output variable, and then take the average of the relative error of different output variables, counted as the total relative error, and finally determine the bounds of relative error of each prediction model.

The individual relative error:

(20) $$e_{r}(x)=\frac{\left|x^{*}-x\right|}{x}$$

The mean of relative error for each output variable:

(21) $$\bar{e_{r}(x)}=\frac{\sum_{1}^{i}\frac{\left|x_{i}^{*}-x\right|}{x}}{i}$$

The total relative error:

(22) $$\frac{{\sum_{1}^{j}\bar{e_{r}(x)}}_{j}}{j}$$

The relative error bound:

(23) $$\varepsilon^{+}\geq \frac{{\sum_{1}^{j}\bar{e_{r}(x)}}_{j}}{j}\geq \varepsilon^{-}$$

The individual relative error refers to taking the absolute value of the relative error of each prediction of the experimental data; the mean of relative error for each output variable refers to the average of the relative errors of the prediction model for each output variable; the total relative error mean refers to the average of the relative errors of each predictive model for all output variables; and the relative error bounds indicate the range of prediction accuracy for each model.

x*—projected value;

*x*—real value;

*i*—the number of groups for each output variable, here taken as 9;

*j*—the number of output variables for each model, here taken as 6;

*ε*^+^—the upper bound of the relative error, which indicates the maximum value of the mean of the total relative error;

*ε^−^*—the lower bound of relative error, which indicates the minimum value of the mean of the total relative error.

**Figure 16:**
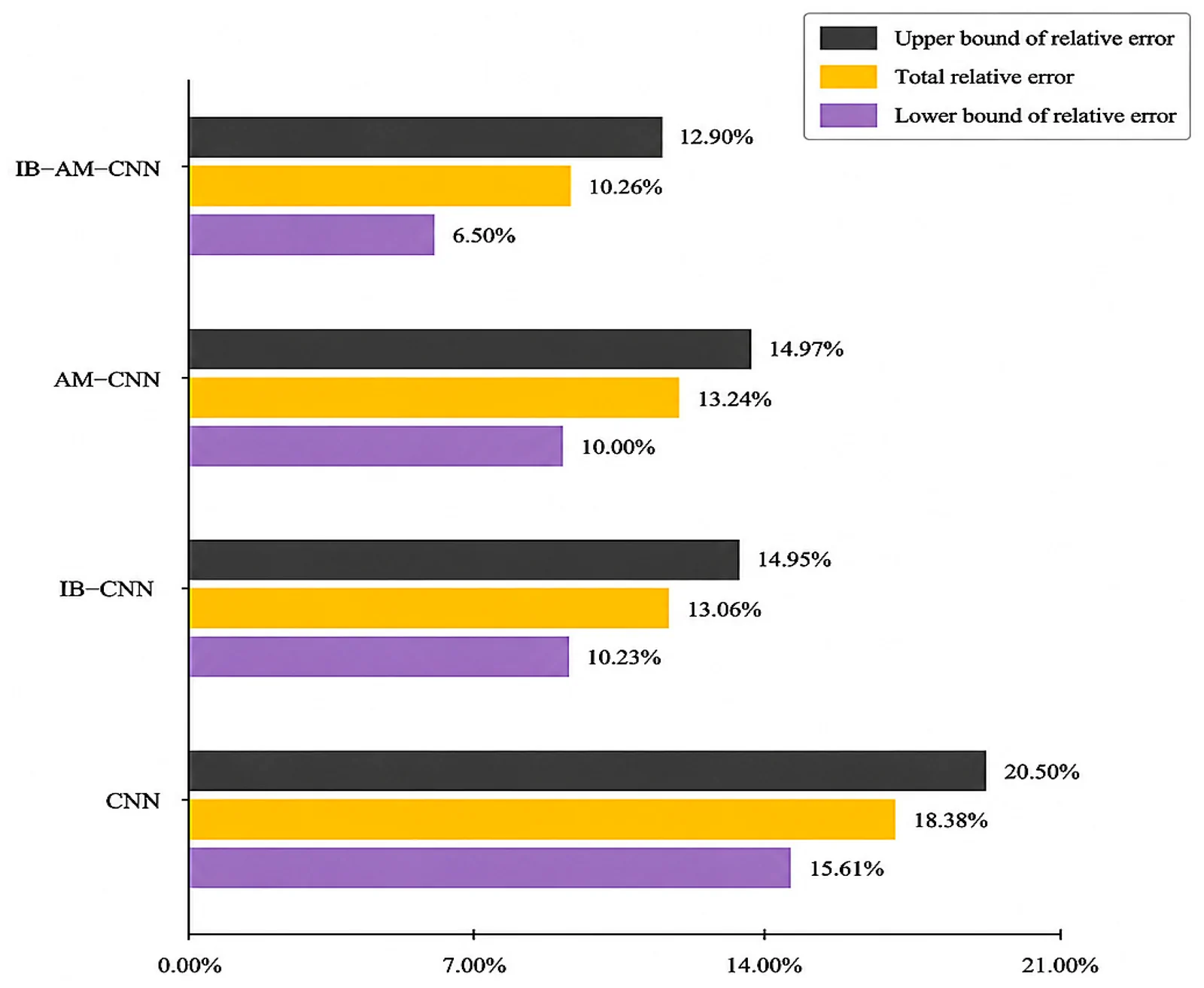
Error analysis diagram of each model.

Calculate the total relative error for each model to get the total relative error and bounds of relative error table, as highlighted in Figure 16.

Figure 16 shows that: CNN prediction error is between 15.61% and 20.50%, and the average prediction error is 18.38%; IB-CNN prediction error is between 10.23% and 14.95%, the average prediction error is 13.06%, and the optimization effect is close to 1/3; AM-CNN prediction error between 10.00% and 14.97%, and the average prediction error is 13.24% the optimization effect is close to 1/3; and IB-AM-CNN prediction error is between 6.50% and 12.90%, with an average prediction error of 10.26%, which improves the optimization effect to 44% compared to the original CNN model. It is clear that both the information bottleneck theory and the attention theory can optimize the CNN model used for slurry prediction, and both of their prediction accuracies are better improved, with the IB-AM-CNN with the highest accuracy using the dual optimization of the information bottleneck theory and the attention theory.

## 4. Discussion

Somewhere in China, a highway roadbed reinforcement project, due to the loess facing strong wet subsidence, encountered slope sliding, pavement settlement, and other problems in the operation process of some sections of the roadbed, resulting in the formation of several longitudinal cracks in the road surface. These issues tend to continue developing in the affected sections of the highway, posing safety hazards. Therefore, grouting reinforcement was carried out for foundation treatment at this site. In addition, the above analysis of the grouting effectiveness was conducted in this project, and the assessment standards for the various factors required by the project are shown in Table 4.

In conjunction with Table 2, the slurry ratio groupings of Table 1a were screened and the groups that met the engineering requirements of Table 4 are shown in Table 5. These four sets of feasible mix proportions also validated the reasonableness of the feasible region identified by the subsequent grid search; that is, all mix proportions from the orthogonal experiments that met the engineering requirements fell within the range of feasible parameters identified by the grid search.

To determine the recommended mix design, this study employed a grid search method in a three-dimensional parameter space. Using the water-to-binder ratio (1.0:1 to 1.5:1, with a step size of 5%), fly ash dosage (10% to 50%, with a step size of 5%), and water glass volumetric dosage (1% to 10%, with a step size of 1%) as search variables, a total of 990 sets of candidate mix designs were generated. Each mix design was input into a pre-trained IB-AM-CNN model to predict six performance metrics. The engineering evaluation criteria in Table 4 were used as constraints to screen the feasible region. Among the candidate mix designs that satisfied all constraints, the one with the best overall performance was selected based on the decision criteria of the highest compressive strength and the highest stone rate.

It should be noted that the recommended mixture, with a water-to-binder ratio of 1.25:1, 20% fly ash dosage, and 10% water glass volumetric dosage, was not included in the nine orthogonal test groups listed in Table 1a. Instead, it was obtained through model interpolation within the investigated parameter range. Therefore, this mixture should be regarded as a model-derived recommended design rather than an experimentally determined optimum. The engineering applicability of this recommended mixture was further evaluated through field loading tests.

**Figure 17:**
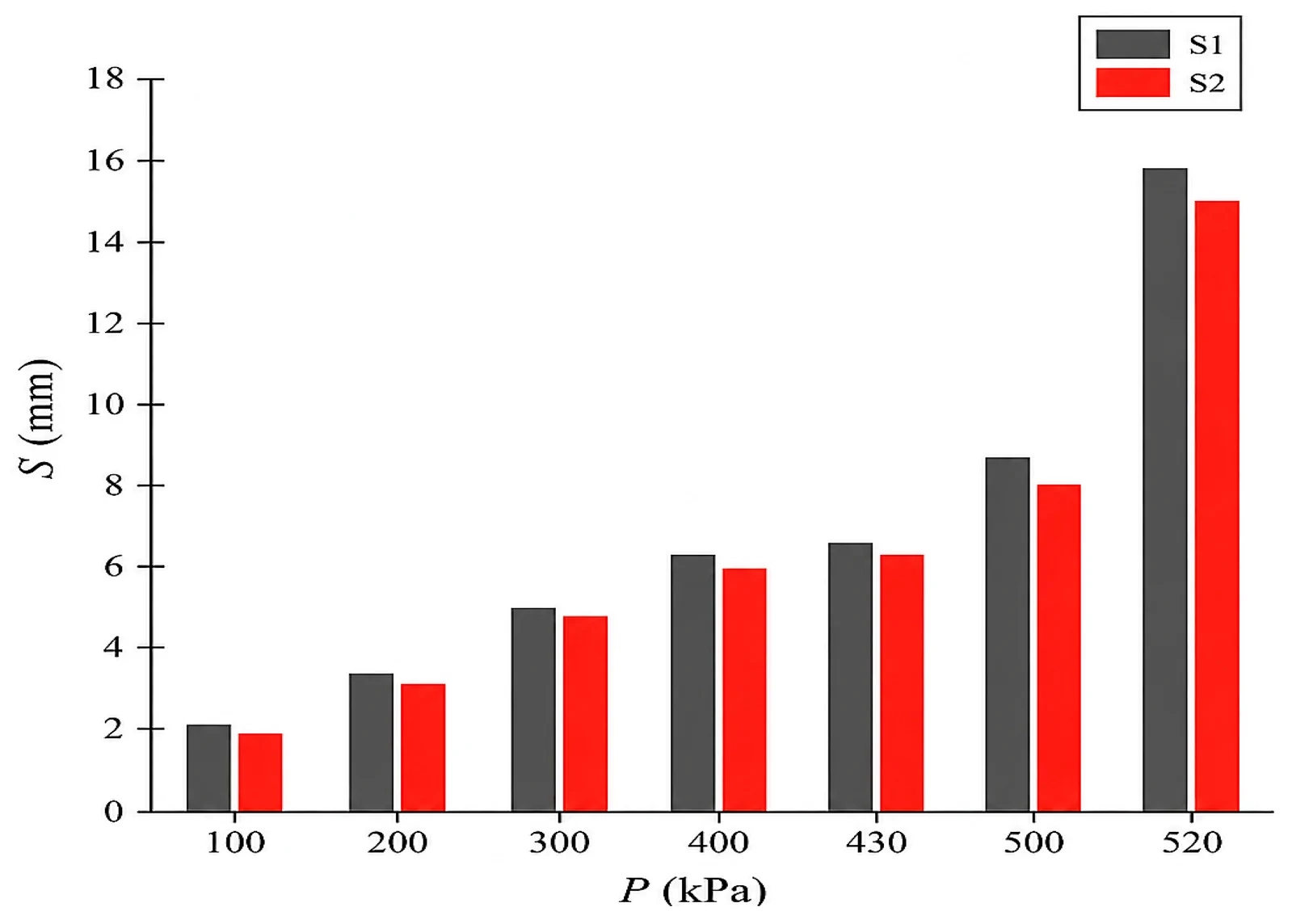
Load-settlement curve of single pile composite subgrade.

It should be noted that the validation method used in this study combines laboratory testing with engineering practice: (1) Laboratory performance validation (Table 6)—all six properties of the new grout were tested using the same method as in the orthogonal experiments based on the optimal mix design to verify the predictive accuracy of the IB-AM-CNN model. (2) Engineering application validation—the optimal grout was used for on-site grouting and reinforcement of a loess subgrade to confirm the applicability of the mix design recommended by the model under actual engineering conditions. In order to further verify the grouting effect of this group of slurry, the improved slurry with this ratio was used to carry out the field loading test of two monopile composite roadbeds, and the load-settlement curves of the monopile composite roadbeds are shown in Figure 17, in which s_1_ and s_2_ denote two different monopiles, and the final settlement of the two monopiles in 70 min was 16 mm, which were all in line with the engineering requirements, indicating that the modified slurry mix proportion selected based on the model predictions can better meet the demands of actual engineering projects.

Most existing studies on the performance prediction of cement-based materials focus solely on compressive strength, employing methods such as artificial neural networks, Random Forests, and deep learning, with average absolute percentage errors typically ranging from 8% to 15%. In this study, targeting fly ash grout, we developed a multi-output CNN model optimized using the information bottleneck–attention mechanism, capable of simultaneously predicting six parameters: slurry density, viscosity, initial setting time, final setting time, compressive strength, and stone rate. Experimental validation showed that the model’s average prediction error was 10.26%. This study overcomes the limitations of single-output compressive strength prediction and provides valuable insights for future machine learning research in the field of cement-based cementitious materials.

This study still has the following limitations: (1) The input variables consider only three factors—water-to-binder ratio, fly ash dosage, and water glass volumetric dosage—and do not include parameters that significantly affect slurry performance, such as curing temperature, age, cement type, and fineness; consequently, the model’s scope of application is limited. (2) The CNN model is a data-driven “black-box” model that lacks an explicit representation of the hydration reaction mechanisms and physical processes of the grout, resulting in insufficient interpretability of the prediction results. (3) The engineering validation of the optimal mix design was limited to a specific scenario involving grouting reinforcement of loess subgrades; its applicability under other geological conditions and for other types of engineering projects requires further verification.

## 5. Conclusions

In order to study the relationship between the proportion and performance of fly ash slurry, establish a performance prediction model and apply it to engineering, this paper uses convolutional neural network to predict it, and optimizes the neural network by using the bottleneck theory and the attention mechanism, establishes a multi-objective performance prediction model for fly ash slurry, and compares the superiority of IB-AM-CNN model in predicting the performance of the slurry in terms of the theory and model data. The specific conclusions are as follows:

(1) By integrating information bottleneck theory and attention mechanisms into the CNN, the proposed IB-AM-CNN model achieved improved five-fold cross-validation performance and lower prediction errors than the baseline CNN within the investigated mix proportion range, suggesting better generalization performance under the present dataset.
(2) With the trained present model, only three input variables, water-to-binder ratio, fly ash dosage and water glass volumetric dosage, are required to predict four slurry properties and two properties of the corresponding stone bodies for this slurry.
(3) The results indicate that both information bottleneck theory and attention theory optimize the convolutional neural network used for predicting the performance of fly ash slurry. Their optimization effect is around 1/3, i.e., the prediction errors is between 10.00% and 14.97%. The average prediction error is 13.15%, whereas optimization of the CNN model by using both information bottleneck theory and attention theory can be improved to 44%, i.e., the prediction errors is between 6.5% and 12.9%, and the average prediction error is 10.26%.
(4) By considering the required performance of the slurry and stone body for engineering applications, the IB-AM-CNN model recommended a mixture ratio of 1.25:1 water-to-binder ratio, 20% fly ash dosage, and 10% water glass volumetric dosage within the investigated parameter range. This recommended mixture exhibited satisfactory engineering applicability during on-site loading tests.

## Figures and Tables

**Table 1:** (**a**) Orthogonal experimental design; (**b**) range analysis of orthogonal test results.

(**a**)							
**Number**	**Water-to-Binder Ratio**	**Fly Ash Dosage**	**W** **ater Glass** **Volumetric Dosage**	**Cement (g)**	**Fly Ash (g)**	**Water (g)**	**Water Glass Solution (g)**
1	1.0:1	10%	1%	900	100	994	10
2	1.0:1	30%	10%	700	300	936	100
3	1.0:1	50%	5%	500	500	968	50
4	1.25:1	10%	10%	900	100	1186	100
5	1.25:1	30%	5%	700	300	1218	50
6	1.25:1	50%	1%	500	500	1244	10
7	1.5:1	10%	5%	900	100	1468	50
8	1.5:1	30%	1%	700	300	1494	10
9	1.5:1	50%	10%	500	500	1436	100
(**b**)							
**Performance Indicator**		**R_A_ (** **Water-to-Binder Ratio** **)**	**R_B_ (** **Fly Ash Dosage** **)**	**R_C_ (W** **ater Glass Volumetric Dosage** **)**			**Primary/Secondary Order**
Slurry density		0.163	0.007	0.067			ACB
Viscosity		9.000	12.667	26.000			CBA
Initial setting time		253.333	160.000	10.000			ABC
Final setting time		456.667	486.667	86.667			BAC
Compressive strength		3.660	0.770	0.733			ABC
Stone rate		17.667	4.000	14.333			ACB

Note: R_A_, R_B_, and R_C_ represent the ranges of the water-to-binder ratio, fly ash dosage, and water glass volumetric dosage, respectively. A larger range indicates that the corresponding factor has a greater impact on the performance index.

**Table 2:** Experimental results of slurry performance.

Number	Slurry Density (g/cm^3^)	Viscosity (Pa·s)	Initial Setting Time (min)	Final Setting Time (min)	Compressive Strength (MPa)	Stone Rate/%
1	1.58 ± 0.017	26 ± 2.0	1240 ± 28	1750 ± 46	1.50 ± 0.07	85 ± 2.0
2	1.65 ± 0.017	40 ± 2.6	1300 ± 36	2030 ± 50	3.01 ± 0.11	100 ± 0
3	1.61 ± 0.017	29 ± 2.0	1380 ± 40	2140 ± 56	4.11 ± 0.17	92 ± 2.0
4	1.54 ± 0.017	33 ± 2.0	1350 ± 30	2100 ± 50	4.10 ± 0.15	98 ± 1.7
5	1.51 ± 0.010	20 ± 1.0	1460 ± 43	2350 ± 60	3.80 ± 0.15	88 ± 2.0
6	1.47 ± 0.010	19 ± 1.0	1510 ± 40	2590 ± 70	5.20 ± 0.24	77 ± 2.0
7	1.45 ± 0.017	17 ± 1.0	1480 ± 40	2200 ± 50	7.30 ± 0.28	75 ± 1.0
8	1.42 ± 0.010	16 ± 1.0	1540 ± 50	2310 ± 60	6.40 ± 0.23	71 ± 2.0
9	1.48 ± 0.017	66 ± 4.6	1660 ± 60	2780 ± 80	5.90 ± 0.26	78 ± 1.0

Note: Values are expressed as mean ± standard deviation (SD) based on three replicate measurements (*n* = 3). The standard deviation was calculated directly from the individual experimental measurements.

**Table 3:** Five-fold cross-validation performance for each output variable in the original physical units.

Output Variable	CNN	IB-CNN	AM-CNN	IB-AM-CNN
Slurry density (g/cm^3^)	0.86/0.083/0.064	0.90/0.069/0.053	0.92/0.061/0.046	0.96/0.043/0.032
Viscosity (Pa·s)	0.78/8.80/6.90	0.84/7.40/5.60	0.88/6.30/4.80	0.94/4.60/3.40
Initial setting time (min)	0.82/168/128	0.87/142/106	0.90/126/93	0.95/88/65
Final setting time (min)	0.80/245/187	0.85/211/160	0.89/180/136	0.94/132/98
Compressive strength (MPa)	0.84/0.91/0.70	0.88/0.75/0.58	0.91/0.64/0.49	0.96/0.45/0.34
Stone rate (%)	0.82/6.80/5.20	0.88/5.50/4.10	0.90/4.80/3.60	0.95/3.40/2.50
Mean (R^2^)	0.82	0.87	0.90	0.95

Note: Each cell in the table is arranged in the following order: R^2^/RMSE/MAE. In addition, the output variables are defined as follows: convolutional neural network (CNN); information bottleneck theory optimized convolutional neural network (IB-CNN); attention mechanism optimized convolutional neural network (AM-CNN); information bottleneck-attention optimized convolutional neural network (IB-AM-CNN).

**Table 4:** Engineering evaluation standards.

Engineering Performance	Performance Indicators	Criteria for Evaluation
Load bearing capacity	Compressive strength	Not less than 3 MPa
Hydrophobicity	Stone rate	Greater than 75 percent
Rheology	Viscosity	22 Pa·s~29 Pa·s
Controllability	Initial setting time	Less than 1500 min
	Final setting time	More than 2000 min

**Table 5:** Groups that meet engineering needs.

Number	Water-to-Binder Ratio	Fly Ash Dosage	Water Glass Volumetric Dosage
3	1.0:1	50%	5%
5	1.25:1	30%	5%
6	1.25:1	50%	1%
7	1.5:1	10%	5%

**Table 6:** Comparison of predicted values from the optimal mix design model with experimental measurements.

Performance Indicator	Model Predicted Value	Experimental Measured Value	Relative Error (%)
Slurry density(g/cm^3^)	1.52	1.50 ± 0.02	1.32
Viscosity (Pa·s)	28	30 ± 2	6.67
Initial Setting Time (min)	1400	1430 ± 40	2.10
Final Setting Time (min)	2200	2150 ± 60	2.27
Compressive Strength (MPa)	4.50	4.20 ± 0.25	7.14
Stone Rate (%)	95	93 ± 2	2.11

## Data Availability

The original contributions presented in the study are included in the article. Further inquiries can be directed to the corresponding author.

## References

[1] Wang N., Sun X., Zhao Q., Yang Y., Wang P. (2020). Leachability and adverse effects of coal fly ash: A review. J. Hazard. Mater..

[2] Luo Y., Wu Y., Ma S., Zheng S., Zhang Y., Chu P.K. (2021). Utilization of coal fly ash in China: A mini-review on challenges and future directions. Environ. Sci. Pollut. Res..

[3] Montgomery A., Kasaniya M., Zhao P., Thomas M., Peterson K. (2024). The dam that fly ash built. J. Microsc..

[4] Fernando S., Gunasekara C., Law D.W., Nasvi M.C.M., Setunge S., Dissanayake R. (2021). Life cycle assessment and cost analysis of fly ash-rice husk ash blended alkali-activated concrete. J. Environ. Manag..

[5] El Mendili Y., Bouasria M., Benzaama M.H., Khadraoui F., Le Guern M., Chateigner D., Gascoin S., Bardeau J.F. (2021). Mud-Based Construction Material: Promising Properties of French Gravel Wash Mud Mixed with Byproducts, Seashells and Fly Ash as a Binder. Materials.

[6] Xiang H., Zhen W., Ke Y., Xiang Y., Shuxin H., Yihao S., Bin L. (2023). Experimental Study on the Diffusion Law of Coal-Based Solid Waste Slurry with Grouting Filling in the Gangue Voids of the Caving Zone. ACS Omega.

[7] Huo W., Zhu Z., Zhang J., Kang Z., Pu S., Wan Y. (2021). Utilization of opc and fa to enhance reclaimed lime-fly ash macadam based geopolymers cured at ambient temperature. Constr. Build. Mater..

[8] Ashfaq M.H., Sharif M.B., Irfan-Ul-Hassan M., Sahar U.U., Akmal U., Mohamed A. (2024). Up-scaling of fly ash-based geopolymer concrete to investigate the binary effect of locally available metakaolin with fly ash. Heliyon.

[9] Hwang S., Yeon J. (2022). Fly ash-added, seawater-mixed pervious concrete: Compressive strength, permeability, and phosphorus removal. Materials.

[10] Wasil M., Zabielska-Adamska K. (2022). Tensile Strength of Class F Fly Ash and Fly Ash with Bentonite Addition as a Material for Earth Structures. Materials.

[11] Li W.J., Feng P., Liu X., Xie H., Li D.N. (2026). Construction of an explicit prediction formula for concrete carbonation depth based on interpretable machine learning. J. Chin. Ceram. Soc..

[12] Zhang Z.Q., Du W.J., Liu Y., Ma S.L., Liu X.M., Jing R. (2026). Performance prediction and interpretability analysis of small-sample ultra-high-performance concrete based on machine learning. J. Chin. Ceram. Soc..

[13] Liu X., Li Y.X., Tang S.J., Wang S.M., Xiao Y.Z., Huang Z.H., Cui S.P. (2026). Research progress in predicting the performance of low-carbon cementitious materials using machine learning and deep learning. J. Chin. Ceram. Soc..

[14] Yeh I.C. (1998). Modeling of strength of high-performance concrete using artificial neural networks. Cem. Concr. Res..

[15] Wang X.Y. (2023). Optimization design of low-carbon hybrid concrete containing slag and limestone powder. Environ. Sci. Pollut. Res..

[16] Wang X.Y. (2019). Optimal Design of the Cement, Fly Ash, and Slag Mixture in Ternary Blended Concrete Based on Gene Expression Programming and the Genetic Algorithm. Materials.

[17] Lee S.C. (2003). Prediction of concrete strength using artificial neural networks. Eng. Struct..

[18] Ziolkowski P., Niedostatkiewicz M. (2019). Machine Learning Techniques in Concrete Mix Design. Materials.

[19] Wang M., Du M., Jia Y., Chang C., Zhou S. (2024). Carbon Emission Optimization of Ultra-High-Performance Concrete Using Machine Learning Methods. Materials.

[20] Liu Y. (2022). High-Performance Concrete Strength Prediction Based on Machine Learning. Comput. Intell. Neurosci..

[21] Xu J., Zhou L., He G., Ji X., Dai Y., Dang Y. (2021). Comprehensive Machine Learning-Based Model for Predicting Compressive Strength of Ready-Mix Concrete. Materials.

[22] Huang X., Sresakoolchai J., Qin X., Ho Y.F., Kaewunruen S. (2022). Self-Healing Performance Assessment of Bacterial-Based Concrete Using Machine Learning Approaches. Materials.

[23] Xu Y., Ahmad W., Ahmad A., Ostrowski K.A., Dudek M., Aslam F., Joyklad P. (2021). Computation of High-Performance Concrete Compressive Strength Using Standalone and Ensembled Machine Learning Techniques. Materials.

[24] Javed M.F., Fawad M., Lodhi R., Najeh T., Gamil Y. (2024). Forecasting the strength of preplaced aggregate concrete using interpretable machine learning approaches. Sci. Rep..

[25] Wang M., Kang J., Liu W., Su J., Li M. (2022). Research on prediction of compressive strength of fly ash and slag mixed concrete based on machine learning. PLoS ONE.

[26] Rajczakowska M., Szeląg M., Habermehl-Cwirzen K., Hedlund H., Cwirzen A. (2023). Interpretable Machine Learning for Prediction of Post-Fire Self-Healing of Concrete. Materials.

[27] Xiao J., Ye H., He X., Zhang H., Wu F., Chua T.S. Attentional factorization machines: Learning the weight of feature interactions via attention networks. Proceedings of the 26th International Joint Conference on Artificial Intelligence (IJCAI-17).

[28] Chen J., Zhang H., He X., Nie L., Liu W., Chua T.S. Attentive collaborative filtering: Multimedia recommendation with item- and component-level attention. Proceedings of the 40th International ACM SIGIR Conference on Research and Development in Information Retrieval (SIGIR ’17).

[29] Cheng Z., Ding Y., He X., Zhu L., Kankanhalli M. A^3NCF: An Adaptive Aspect Attention Model for Rating Prediction. Proceedings of the 27th International Joint Conference on Artificial Intelligence (IJCAI-18).

[30] Test Method of Cement Mortar Strength (ISO Method). Standardization Administration of China: Beijing, China, 2021.

[31] Technical Specification for Grouting. Ministry of Industry and Information Technology of the People’s Republic of China: Beijing, China, 2018.

[32] Technical Specification for Cement Grouting Construction of Hydraulic Buildings. Ministry of Water Resources of the People’s Republic of China: Beijing, China, 2020.

[33] Cementitious Grout. Ministry of Industry and Information Technology of the People’s Republic of China: Beijing, China, 2018.

[34] Slonim N., Tishby N. (2000). Agglomerative information bottleneck. Advances in Neural Information Processing Systems 12, Proceedings of the (NIPS 1999), Denver, CO, USA, 29 November–4 December 1999.

[35] Tishby N., Zaslavsky N. Deep learning and the information bottleneck principle. Proceedings of the 2015 IEEE Information Theory Workshop (ITW 2015).

[36] Shwartz-Ziv R., Tishby N. (2017). Opening the black box of deep neural networks via information. arXiv.

